# Atmospheric methane consumption in arid ecosystems acts as a reverse chimney and is accelerated by plant-methanotroph biomes

**DOI:** 10.1093/ismejo/wraf026

**Published:** 2025-03-02

**Authors:** Nathalie A Delherbe, Oscar Gomez, Alvaro M Plominsky, Aaron Oliver, Maximino Manzanera, Marina G Kalyuzhnaya

**Affiliations:** Department of Biology, San Diego State University, San Diego, CA 92129, United States; Department of Biology, San Diego State University, San Diego, CA 92129, United States; Marine Biology Research Division, Scripps Institution of Oceanography, University of California San Diego, La Jolla, CA 92037, United States; Marine Biology Research Division, Scripps Institution of Oceanography, University of California San Diego, La Jolla, CA 92037, United States; Institute for Water Research and Department of Microbiology, University of Granada, Granada 18071, Spain; Department of Biology, San Diego State University, San Diego, CA 92129, United States

**Keywords:** methanotrophs, arid soil, desert microbiomes, *Methylocaldum*, plants, and methane cycle

## Abstract

Drylands cover one-third of the Earth’s surface and are one of the largest terrestrial sinks for methane. Understanding the structure–function interplay between members of arid biomes can provide critical insights into mechanisms of resilience toward anthropogenic and climate-change-driven environmental stressors—water scarcity, heatwaves, and increased atmospheric greenhouse gases. This study integrates *in situ* measurements with culture-independent and enrichment-based investigations of methane-consuming microbiomes inhabiting soil in the Anza-Borrego Desert, a model arid ecosystem in Southern California, United States. The atmospheric methane consumption ranged between 2.26 and 12.73 μmol m^2^ h^−1^, peaking during the daytime at vegetated sites. Metagenomic studies revealed similar soil-microbiome compositions at vegetated and unvegetated sites, with *Methylocaldum* being the major methanotrophic clade. Eighty-four metagenome-assembled genomes were recovered, six represented by methanotrophic bacteria (three *Methylocaldum*, two *Methylobacter,* and uncultivated *Methylococcaceae*). The prevalence of copper-containing methane monooxygenases in metagenomic datasets suggests a diverse potential for methane oxidation in canonical methanotrophs and uncultivated Gammaproteobacteria. Five pure cultures of methanotrophic bacteria were obtained, including four *Methylocaldum*. Genomic analysis of *Methylocaldum* isolates and metagenome-assembled genomes revealed the presence of multiple stand-alone methane monooxygenase subunit C paralogs, which may have functions beyond methane oxidation. Furthermore, these methanotrophs have genetic signatures typically linked to symbiotic interactions with plants, including tryptophan synthesis and indole-3-acetic acid production. Based on *in situ* fluxes and soil microbiome compositions, we propose the existence of arid-soil reverse chimneys, an empowered methane sink represented by yet-to-be-defined cooperation between desert vegetation and methane-consuming microbiomes.

## Introduction

Greenhouse gases (GHGs) are the chemical footprint of natural and anthropogenic activities that accelerate climate change. Methane (CH_4_) is the second most abundant GHG, after carbon dioxide (CO_2_), constituting the most abundant reduced compound [1] and hydrocarbon [2] in our atmosphere. The global warming potential of CH_4_ is 84 times higher than CO_2_ over a period of 20 years [3]. Approximately 500–600 Tg (1Tg = 10^12^ g) of CH_4_ are emitted globally into the atmosphere every year from different natural and anthropogenic processes [4–9], posing an urgent need for worldwide mitigation efforts [10]. Biological CH_4_ emissions are driven by the interplay of two groups of organisms: CH_4_ producers (mostly methanogens) and CH_4_ consumers (often described as methanotrophs). The metabolic activities of these two functional microbial groups determine the net methane flux of ecosystems as a CH_4_ source (presenting net emissions) or sink (presenting a net uptake from the atmosphere) [4]. Soils are the major sinks of atmospheric CH_4,_ consuming 30–42 Tg per year through the activity of methanotrophic bacteria [11–15]. CH_4_ uptake rates in soils—which vary significantly depending on the ecosystem—have declined in the past decades, mostly due to anthropogenic disturbances [16–18]. The CH_4_ consumption rates in dryland areas (which include semiarid, arid, and hyper-arid regions) have average annual consumption rates as high as 0.66 mg CH_4_ m^−2^ d^−1^ [19], which is comparable to that of grassland and forest soils (0.65 mg CH_4_ m^−2^ d^−1^ and 0.74–1.26 mg CH_4_ m^−2^ d^−1^, respectively) [20, 21]. The fact that dryland environments comprise roughly one-third of the land surface on Earth [22, 23] denotes the importance of studying these regions for correct modeling of the global CH_4_ budget. As dryland microbes contribute to global climate regulation through CO_2_, reactive N, and CH_4_ emissions, these processes will also likely alter the rate of GHG release and impact the rate of climate change [24]. Considering that dryland ecosystems are predicted to expand due to climate change and land-use shifts [25], characterization of soil microbial communities, or microbiota, responsible for biogeochemical fluxes from pristine arid soils is essential for understanding the community dynamics across organizational scales and its effects on global carbon fluxes.

Globally, dryland microbiomes are dominated by bacteria members of the phylum Actinomycetota (Actinobacteria), Chloroflexota (Chloroflexi), and the Pseudomonadota (Proteobacteria) [24]. Less is known about microbial groups that control the local flux of CH_4_, including CH_4_-oxidizing bacteria, anaerobic methanotrophic archaea, and methanogenic archaea [26]. Different microbial groups, including members of the genera *Methylocapsa*, *Methylococcus,* and the family *Methylocystaceae* have been identified as possible players in methane cycling in these environments [27]. A study assessing >3400 metagenomes to examine the global patterns of CH_4_ metabolism marker gene abundances in soil (a proxy for the distribution of CH_4_-metabolizing microorganisms), has revealed the existence of latitudinal trends in the global abundances of these microbes [28]. The variations in global abundances of CH_4_-metabolizing microorganisms have been primarily governed by vegetation cover [28], with no clear patterns in the structure and composition of methanotrophic communities [27].

This study investigated the role of soil microbiota in modulating CH_4_ fluxes for comprehending deserts as CH_4_ sinks. The Anza-Borrego Desert State Park (mentioned hereafter as “Anza-Borrego”) was selected as a model system for investigating the methanotrophic soil communities, including those inhabiting plant rhizosphere. The Anza-Borrego Desert ecosystem lies within the Colorado Desert of southern California, United States. Once a tropical forest, then a wetland and savanna, and finally one of the hottest deserts in the western United States, this area was assessed to determine the impact of microbiota from semiarid regions on the methane cycle. This study integrates *in situ* CH_4_ fluxes measurements, microbial metagenomics, and the metabolic potential of methanotrophic bacteria isolates, thus providing insights into how the soil microbiota, with and without vegetation, consumes CH_4_.

## Materials and methods

### Methane flux measurements

Methane flux measurements at the Anza-Borrego Desert State Park (33.305667, −116.254626; at 208 m over sea level; Supplementary Fig. S1A) were conducted using an ultra-portable greenhouse gas analyzer (UPGGA) LGR model 915–001 (ABB Inc., Quebec, Canada). The initial data, collected in 2016 and 2018 using a 3 L chamber, suggested a positive correlation between soil cover (plants) and methane consumption rates. We returned in March 2020 and 2023 to remeasure methane fluxes using a larger 12.87 L chamber, placed over either vegetated (with the endemic desert verbena *Abronia villosa*) or unvegetated patches (Supplementary Fig. S1B-C). Measurements taken every 1 s were collected during >15 min on six sites spaced >3 m apart. On each site, vegetated and unvegetated patches located <0.5 m from each other were measured (Supplementary Fig. S1D-E).

### Soil samples for metagenomics

Two different sampling campaigns took place in 2016 and 2023. In February and May 2016, samples were collected from three depths of 5, 10, and 20 cm in both vegetated (e.g., brittlebush, desert willow) and unvegetated soil patches. Soil samples from 2016 were placed in sterile bags and kept at ambient temperature. Samples (25 g) for DNA and RNA extraction were collected into 50 ml tubes containing 25 ml of TE buffer with 10% phenol:ethanol (5:95 v/v) stop solution, placed on ice, and transported to the laboratory for processing. Enrichment cultures and DNA extractions were started within 24 h of sample collection. Subsets of collected soil samples (25 g each) were incubated with ^13^CH_4_ added to 1% of headspace every second day for 7 days. The ^13^C-DNA fractions were collected as previously described [29] and submitted for sequencing. Significant cross-feeding was observed in the ^13^C-study experiments, and the data were excluded from analyses provided here; however, the resulting metagenomic sequences are available on the Joint Genome Institute (JGI)–Integrated Microbial Genomes (IMG) website (Supplementary Table S1). From 2016–2018 sequencing efforts, we described only metagenome-assembled genomes (MAGs) in this study. Additional soil samples for metagenomic studies were collected in Spring 2023. In situ methane flux measurements were obtained for each site. Six soil replicates equivalent to 1 ml volumes of soil enclosed in a 2 ml cryovial were collected at 10 cm of depth on five sites (vegetated or unvegetated, Supplementary Fig. S1F). Samples were preserved immediately in dry ice and subsequently stored at −80°C until processed for DNA extraction.

### Enrichment studies, pure culture, and cultivation

Methanotrophic cultures were isolated from soil samples collected in Spring 2015, 2016, and 2018, using the previously described enrichment strategy [30]. All axenic cultures of methanotrophic bacteria were cultivated using P_0%_ medium [31, 32] and maintained at 30°C at constant agitation at 200 rpm. Methylotrophic and non-methylotrophic satellite cultures from methanotrophic enrichments or consortia were achieved by serial plating on diluted minimal medium (0.3x Hypho) [33] supplemented with 0.1% methanol or P_0%_ media supplemented with 0.1% (v/v) methanol (P_M_) and/or 5% Nutrient Broth (P_NB_, Thermo Scientific™ Oxoid™).

### DNA preparation, sequencing, assembly, and annotation

Microbiome DNA was extracted using two different methods. Samples from 2016 were extracted using a modified 25:24:1 phenol/chloroform/isoamyl alcohol protocol [34]. Samples collected in 2023 were extracted using a protocol adapted from Povedano-Prieto et al. [35] and Zeugin and Hartley [36, 37] (Supplementary Material). Samples were sequenced using 2 × 250 chemistry on NovaSeq (Illumina). Metagenomic reads were processed in KBase [38] following their pipeline for processing metagenomic samples [39], using MetaSPAdes for assembly [40]. Assembled metagenomes were submitted to the Integrated Microbial Genomes & Microbiomes (IMG/M) system (Supplementary Table S1) and annotated with the IMG Pipeline (v.5.1.17 [41]).

Genomic DNA was extracted from the isolated bacteria and co-cultures utilizing the GeneJET (Thermo Scientific) or DNeasy (Qiagen) DNA purification kits, following the manufacturer’s protocols. The 2016 metagenome and isolate genome sequencing and assembling were executed by the JGI [41, 42]. All metagenomes and genomes from this study were uploaded and annotated through the IMG annotation pipelines (v.4.15.1, v.4.15.2, or v.4.16.1 [41]) following JGI protocols (Supplementary Tables S1-S2).

### Taxonomic assignment of reads

Quality filtered pair end metagenomic reads were classified using Kaiju (v1.9.0 [43]) with NCBI BLAST nr + euk (10-Mar-2022) [44, 45] as a reference database, allowing mismatches (greedy mode) with 3 maximum mismatches, 65 of minimum Bitscore, and a maximum E-value of 0.01. Kaiju outputs were converted to summary tables agglomerated at the family taxonomic level using the Kaiju2table program (nested inside Kaiju). Individual metagenome tables were merged using R (v4.3.2).

### Alpha and Beta diversity

The resulting merged count, metadata, and taxonomy tables from 2023 metagenomes were further processed using PhyloSeq [46]. The alpha diversity of taxonomically assigned metagenomic reads without any normalization was determined using Breakaway [47]. Count data was rarefied to the smallest read set (using “rarefy_even_depth”) and used to calculate various measures of alpha diversity (using “estimate_richness”) within PhyloSeq. Additionally, community similarity/dissimilarity metrics were calculated using a Bray–Curtis dissimilarity distance matrix with the rarefied datasets and plotted as an Non-metric Multidimensional Scaling (using “ordinate” and “plot_ordination”) with PhyloSeq. Graphs were visualized using ggplot2 (v3.3.6 [48]), and all processing was done in R (v4.3.2).

### Recovery and phylogeny of copper monooxygenases from desert metagenomes

Translated sequences assigned to KEGGs IDs K10946 (*pmoC/amoC*), K10944 (*pmoA*/*amoA*), K10945 (*pmoB*/*amoB*) encoding the three subunits for the two copper monooxygenases (CuMOs), particulate methane monooxygenase (*pmoCAB*), and ammonia monooxygenase (*amoCAB*), were retrieved from the Anza-Borrego 2023 metagenomes annotated in IMG/M. All sequences from publicly available prokaryote genomes on the IMG/M database (v2023–10) for these genes were also retrieved, excluding MAGs without detailed source information. Available MAGs for NC10 and endosymbionts were included in the analysis. A phylogenetic tree for each subunit was generated including both sets of genes (Anza-Borrego 2023 metagenomes and publicly available). All phylogenetic analyses of this study were performed by making an initial alignment using the multiple alignment program MAFFT (v7.511), choosing the E-INS-i method [20, 21]. Phylogenetic relationships were inferred with the MEGA X software (v10.2.6) using the maximum likelihood method. The model of each gene sequence set with the lowest Bayesian information criterion (BIC) scores was calculated utilizing partial deletion with a site coverage cutoff of 95% and corresponded to GTR plus gamma distribution with invariant sites (GTR + G + I) [49–52]. A total of 500 bootstrap replications were computed for each phylogenetic tree of this study. The Newick output of the phylogeny was further processed in iTol [53] to color-code the position of each metagenome-retrieved sequence, distinguishing between vegetated or unvegetated origin. Additionally, three types of CuMOs (methane, ammonium, or alkane monooxygenases) were color-coded based on previous reports for the substrate each organism utilized with their corresponding CuMOs.

A maximum likelihood tree representing the phylogenetic relationship of the multiple particulate methane monooxygenase subunit C (*pmoC*) sequences of *Methylocaldum* was generated based on their amino acid sequences. The model with the lowest BIC scores for the translated *pmoC* gene set corresponded to LG with a discrete Gamma distribution with five rate categories [49–52]. This analysis involved 40 amino acid sequences. There was a total of 315 positions in the final dataset. A total of 500 bootstrap replications were computed. The genomic contexts were obtained from NCBI GenBank annotations and manually curated and re-annotated using the software Geneious Prime 2023. Exported gene cluster images were manually matched with the corresponding sequences on the PmoC phylogenetic tree.

### Generation of metagenome-assembled genomes

For the 2016 metagenomes, MAGs were generated, quality assessed, and taxonomically assigned on IMG/M with MetaBAT (v0.32.5 [54]), CheckM (v1.0.11 [55]), and GTDB database release 86, GTDB-tk (v0.1.6 [39]), respectively. For the 2023 samples, MAGs were generated in KBase with MetaBAT2 (v1.7 [56]) with a minimum contig length set at 1500 bp. Bins were filtered by quality using CheckM (v1.0.18 [55]), and then taxonomically classified with the GTDB-Tk (v2.3.2 [39]) overwriting taxonomy and selecting the r214 GTDB version. A comparison based on the annotated predicted coding sequences for MAGs from 2016 and 2023 metagenomes was obtained in KBase using DRAM [57].

### In silico reconstruction of *Methylocaldum* PmoC folds

Predicted 3D (tertiary) structures for *Methylocaldum* PmoC proteins were generated using ColabFold (v5.1.2 [58]), and its models from AlphaFold (v2.3.1 [59]). Structure predictions were evaluated based on similarity to the nearest relative with a characterized structure, a particulate MMO (pMMO) enzyme from *Methylococcus capsulatus* strain Bath determined using cryo-electron microscopy (PBD: 7S4J) [60]. Predicted structures were visualized with the software ChimeraX (v1.6.1 [61]), and distances between *Methylocaldum* PmoC predicted structures and *M. capsulatus* pMMO were determined using the ChimeraX command matchmaker.

### In silico evaluation of the metabolic potential of *Methylocaldum* isolates and MAGs

An overall comparison of the metabolic potential for the isolate genomes was performed based on the percentage of genes assigned to each of the 24 COG categories from the IMG/MER annotations [42, 62]. A detailed cross-comparison between the genomes of the isolated strains was done following the established pangenome analysis [63] using the Anvi’o software (v7 [64]). Genome clustering was generated based on the presence/absence of predicted genes (utilizing their amino acid sequence) and grouping by similarity using the MCL algorithm (inflation of 8 [65] and a Minbit score of 0.8). The clusters were organized based on Euclidean distance and Ward linkage methods. The predicted genes were annotated with DIAMOND using the fast parameter against NCBI’s Clusters of Orthologous Groups (COGs) database [66]. KEGG annotation was obtained for each gene call utilizing the KEGG KOfam database as previously described [67]. The Average Nucleotide Identity multiplied by the Aligned Coverage (ANI × AC) values, called full percent identity in the Anvi’o software, were computed with *anvi-compute-genome-similarity* utilizing PyANI [68] across the **10**  *Methylocaldum* genomes. This parameter was chosen because it reflects the genomic relatedness among genomes better than ANI alone [69].

## Results and discussion

### Reverse chimney of arid soils unfolds: In situ methane consumption rates are accelerated by vegetation and linked to daylight intensity

**Figure 1:**
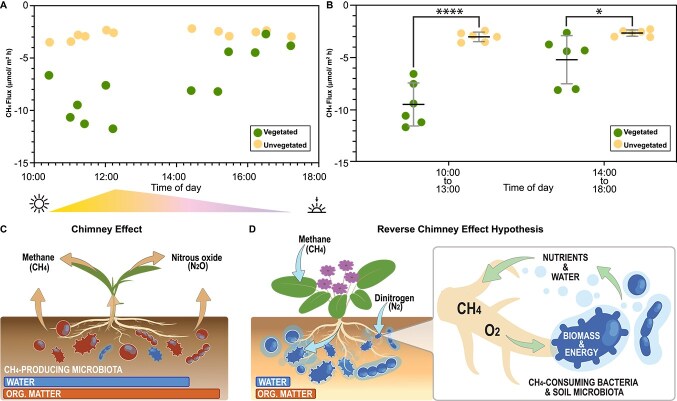
The methane flux (consumption) comparing vegetated and unvegetated patches in the Anza Borrego Desert State Park. (A) the presence of vegetation and methanotrophs correlated positively with an increased consumption rate of methane. (B) Comparison of methane consumption between morning and afternoon. Significance with *P* value <0.0001 (^****^) and 0.0229 (^*^) obtained with unpaired parametric t-test. (C) Graphical summary of the chimney effect. The ribbons in (C) and (D) represent soil moisture (WATER) and organic content (ORG.MATTER). (D) Graphical summary of the proposed reverse chimney effect in arid ecosystems.

Positive correlations between soil cover (vegetation) and methane consumption rates were observed in all field studies conducted in 2016, 2018, 2020, and 2023. The results discussed below are from 2020 and 2023, as they include the most comprehensive set of metadata, such as the type of vegetation—we collected gas fluxes only over *A. villosa* (desert verbena) and the sunlight phase during the measurements. CH_4_ fluxes of Anza-Borrego soil from vegetated sites were observed to vary during the day, reaching their consumption peak during the hours of most intense sunlight with up to 12.73 μmol m^−2^ h^−1^ at noon (Fig. 1A). The average CH_4_ consumption rate in vegetated sites between 10:00 a.m. (i.e., 3 h after sunrise) and 2:00 p.m. (9.07 μmol m^−2^ h^−1^+/− 2.2) was 2.4 times higher than the rates measured 3 h before sunset (3.78 μmol m^−2^ h^−1^+/− 0.81; Fig. 1B). In contrast, unvegetated sites from the immediate vicinity constantly consumed CH_4_ at a rate between 2.26 to 3.74 μmol m^−2^ h^−1^ +/− 0.46 (Fig. 1A). Moreover, the peak CH_4_ consumption rates in vegetated sites between 10:00 a.m. and 2:00 p.m. were 3.17 times higher and significantly different than the average rates for unvegetated sites (*P* value <0.0001; Fig. 1B). This serendipitous observation suggests that Anza-Borrego soil CH_4_ consumption rates at vegetated sites are linked to sunlight intensity, while the consumption rates of unvegetated patches are constant during the day (Fig. 1A). Many terrestrial plants can accelerate methane transfer from soil to atmosphere, a phenomenon often described chimneys of methane (Fig. 1C) [70–73]. Our evidence suggests that arid plants can reverse the methane flow and accelerate atmospheric methane sink. Hence, arid plants and plant biomes will be referred to here as reverse chimneys (Fig. 1D).

### Reverse chimneys of arid soils empowered by vegetation rather than methanotrophic community structure

**Figure 2:**
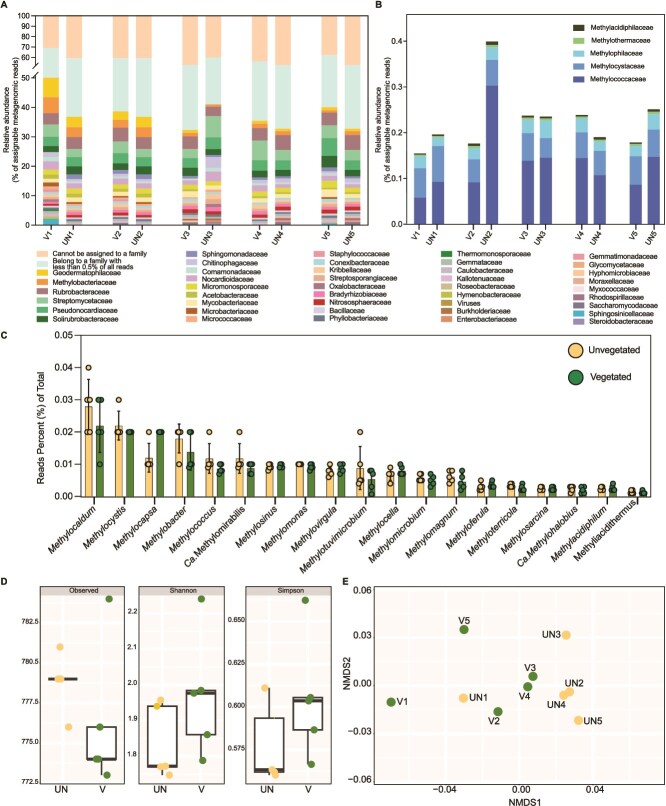
(A) Relative abundance of reads taxonomically assigned groups at a family level. Only abundant families are listed on the legend (>0.5% of assigned reads). (B) Relative abundance of reads taxonomically assigned to methanotrophic groups at family level. (C) Relative abundance of reads taxonomically assigned to methanotrophic genera. (D) Alpha diversity of all normalized taxonomically assignable metagenomic reads of samples with and without vegetation. Non-significant differences were found for Shannon (paired t-test *P* value = 0.184) and Simpson (paired t-test *P* value = 0.2354) indexes. Normalized data was randomly rarefied to 1 721 047 reads. (E) Beta diversity was visualized through a NMDS plot of the Bray–Curtis dissimilarity distances between all the taxonomically assignable metagenomic reads.

The taxonomic assignment of the Anza-Borrego metagenomic reads (microbiome) from 2023 to a (non-viral) family using Kaiju resulted in <45% of the total sequences being assigned, which is within the output range reported for this approach with similar sample types [43]. This assessment revealed that the most abundant members of these microbiomes were similarly distributed among both vegetated and unvegetated sites (Fig. 2A and Supplementary Fig. S2). The most abundant bacteria in these microbiomes belonged to the phyla Actinomycetota (12%–19%), Pseudomonadota (7%–12%), and Acidobacteriota (2%–3%); and to a lesser extent Chloroflexota (1.8%–2.5%), Planctomycetota (1.0%–1.2%), Bacilliota (0.5%–1.1%), Gemmatimonadota (0.8%–1.2%), Bacteroidota (0.5%–2.0%), and Nitrososphaerota (0.4–0.9%). The families Methylobacteriaceae (0.3%–2.4%) and Bradyrhizobiaceae (0.3%–0.5%) were the most abundant Pseudomonadota in these microbiomes (Fig. 2A). Reads assigned to known methanotroph families were found in all vegetated and unvegetated Anza-Borrego 2023 samples. Methylococcaceae had the highest relative abundance of all the methanotrophs detected (representing between 0.06% and 0.3% of all the taxonomically assigned reads), followed by Methylocystaceae (with 0.04%–0.06%), and Methylophilaceae (with 0.01–0.04%). Methylothermaceae and Methylacidiphilaceae were the less abundant ranging between 0.002% and 0.006% (Fig. 2B). Taxonomic assignation at genus level revealed *Methylocaldum* (Methylococcaceae) as the most abundant methanotroph in both vegetated and unvegetated samples, between 0.01–0.04% followed by *Methylocystis* (Methylocystaceae), *Methylocapsa* (Beijerinckiaceae) and *Methylobacter* (Methylococcaceae) with abundances of 0.03–0.09% (Fig. 2C). Reads assigned to methanotrophs were also found in Anza-Borrego 2016 metagenomes, with an average of 0.5% of relative abundance of Methylococcaceae, 0.3% Methylophilaceae, 0.09% of Methylocystaceae, 0.007% Methylothermaceae, and 0.003% Methylacidiphilaceae.

Both normalized and non-normalized approaches, using the taxonomically assignable metagenomic reads, showed no significant differences between the alpha diversity among the Anza-Borrego samples with and without vegetation (Fig. 2D, Supplementary Fig. S2). This trend was also confirmed through pairwise comparison between vegetated and unvegetated when assessing the beta diversity using their normalized taxonomically assignable metagenomic read counts (Fig. 2E) [27].

The similarities between assigned microbial communities do not follow the initial assumption that vegetation, including plant litter and root exudates, serves as a source of organic matter for microbes and leads to distinctive shifts in soil microbial diversity [24]. Since this study investigated the rhizosphere of the seasonal vascular plant *A. villosa* (desert verbena), which has a lifespan of only 2 to 3 months between late autumn and early spring, and samples were collected during the first month of plant growth, it might not produce sufficient exudates to change its rhizosphere microbiota to detectable levels and only influence root epibionts. On the other hand, in nutrient-poor and arid environments, plants do not waste resources on supporting a broader microbiome community but rather tend to control nutrient exchange directly with microbes that colonize roots [74–76]. The results presented here agree with a previous study reporting no significant changes in the total abundance and richness of key marker genes for methanotrophic microbes when assessing different vegetation and climate types from 80 dryland ecosystems [27]. Based on the recovered soil microbiome structures from unvegetated and vegetated sites, we conclude that the observed enhancement of the methane flux is plant-driven and might be achieved in two ways: close colonization of plant roots by *Methylocaldum* spp. or enhanced activity of yet-to-be-discovered methanotrophic functions or organisms.

### Numerous CuMOs are identified in the Anza-Borrego soil metagenomes

**Figure 3:**
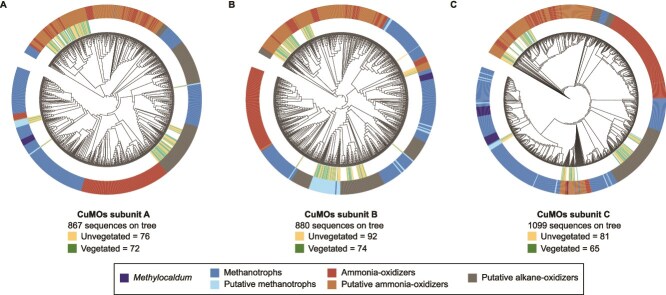
Maximum likelihood trees representing the phylogenetic relationship between CuMOs across bacteria and archaea retrieved from the metagenomes and publicly available genomes. The functional richness of the different subunits from the Anza-Borrego metagenomes is represented by green (vegetated) and yellow (unvegetated) lines at the tips of their corresponding leaves. The outside color-coded ring guides the function of CuMOs, which was assigned based on the taxon at the corresponding leaf. The phylogenetic trees do not show the branch length to facilitate the visualization of clade topology.

Considering the low representation of genomes for dryland soil methanotrophs in current databases and its consequent limitations for their identification in metagenomic datasets, the prevalence of methanotrophs in Anza-Borrego was further assessed using functional metabolic marker genes, such as those coding for the key enzymes for CH_4_ oxidation: methane monooxygenases (MMO). This enzyme has two types: a soluble MMO (sMMO) and pMMO. Only one sMMO sequence was recovered from all Anza-Borrego 2023 metagenomes. The search for pMMO using KEGG and TIGR databases additionally retrieved genes for other CuMOs, representing ammonia and alkane monooxygenases. The phylogenetic relationship of CuMOs retrieved in this functional screening was generated to distinguish which taxa had each of the three possible CuMOs. A total of 148 CuMOs A, 166 CuMOs B, and 146 CuMOs C gene sequences were recovered from the assembled Anza-Borrego 2023 metagenomes (Fig. 3). Thirty-seven and forty-three CuMO sequences associated with canonical methanotrophic taxa (putative methanotrophs) were recovered from vegetated and unvegetated sites, respectively, with the majority corresponding to *pmoB* subunits (25 in vegetated and 27 in unvegetated metagenomes). In addition, CuMOs from ammonia oxidizers were retrieved from both vegetated and unvegetated sites (97 and 116 genes, respectively), as well as CuMOs related to putative hydrocarbon (alkane) oxidizers (58 from vegetated sites and 66 from unvegetated). These results denote the extensive diversity of potential CH_4_ oxidizers in the Anza-Borrego soil microbiomes and suggest that the prevalence of all three types of CuMOs may be independent of the presence of vegetation (Fig. 3).

### MAGs and isolate genomes indicate methanotrophy potential in the Anza-Borrego soil microbiome beyond canonical species

**Figure 4:**
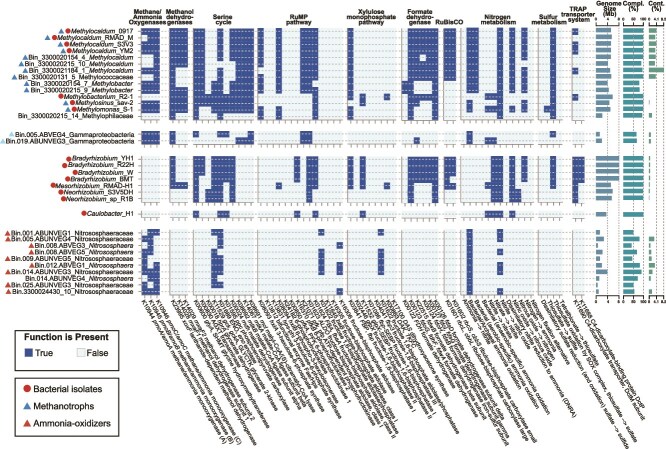
Comparison of the metabolic potential of isolates and relevant MAGs obtained from Anza-Borrego (2016 and 2023). The analysis indicates the presence of key genes involved in relevant pathways for this study.

The 2016 and 2023 Anza-Borrego soil metagenomes were used to generate 84 MAGs, of which 10 were high-quality and 53 medium-quality drafts [77] (Supplementary Fig. S3). Among them, eight MAGs had methane monooxygenase and methanol dehydrogenase genes. These included three *Methylocaldum*, two *Methylobacter*, one *Methylococcaceae*, and two that could only be assigned to the class Gammaproteobacteria, with their *pmoB* subunits having a 36% coverage with 74% identity to *pmoB* of *Methylococcus* and the other a 36% coverage with 75% identity to *Methylocaldum pmoB* (Fig. 4).

Also, other 12 MAGs had at least one of the three CuMO subunit genes (Fig.4): 10 Nitrososphaeraceae (ammonia-oxidizing), (Fig. 4), one Acidimicrobiia, and one Pedosphaerales (Supplementary Fig. S3) with CuMO subunits with high identity to recovered *pmoA* sequences from Anza-Borrego that formed part of the putative alkane-oxidizer clade and *Methylocaldum* stand-alone *pmoC* Types 4 and 5, respectively.

Five pure cultures of methanotrophic bacteria were obtained from the Anza-Borrego cultivation efforts (Fig. 4, Supplementary Table S1). Among these isolates, four corresponded to *Methylocaldum* (strains 0917, S3V3, YM2, and RMAD-M, all with a genome size of 5.2 to 5.4 Mbp), and one corresponded to *Methylosinus* sp. Sav-2 (4.7 Mbp). All isolated *Methylocaldum* grew only when supplemented with methane between 0.04% to 20% (for enrichments and routinary culture, respectively) and did not grow when supplemented with methanol or organic acids as carbon sources. The growth of *Methylosinus* sp. Sav-2 can be supported by methane and methanol. Ten isolates corresponded to non-methanotrophic bacteria, including *Bradyrhizobium* (strains W, R2.2-H, BM-T, Y-H1, all with a genome size of 8.0 Mb), *Neorhizobium* sp. R1-B (5.5 Mb) and S3V5DH (5.8 Mb), *Caulobacter* sp. H1 (3.7 Mb) and the methylotroph *Methylobacterium* sp. R2–1 (5.8 Mb). The growth of methylotrophic cultures can be supported by methanol and organic acids (pyruvate, succinate).

All expected metabolic functions in *Methylocaldum* MAGs were reproduced in at least one of the genomes of isolated strains obtained in this study (Fig. 4, Supplementary Fig. S3). Therefore, since the genus *Methylocaldum* (from the family Methylococcaceae) was the most dominant methanotrophic bacterial group detected in the Anza-Borrego metagenomes (Fig. 2C), further analysis focused on the metabolic potential of the *Methylocaldum* isolates. A genomic comparison between the Anza-Borrego *Methylocaldum* and the 10 *Methylocaldum* genomes (Supplementary Table S3, Supplementary Fig. S4) that were publicly available at the time of the analysis was performed to assess the distinguishing metabolic features of the dryland isolates. For example, all the *Methylocaldum* genomes had the metabolic potential for nitrogen fixation, and both the large (K01601) and small (K01602) subunit of a Type-IA/B ribulose 1,5-bisphosphate carboxylase (Supplementary Information and Supplementary Fig. S5). The Anza-Borrego *Methylocaldum* isolates presented more lanthanide-dependent *xoxF*3-type methanol dehydrogenase paralogs compared to the genomes of isolates from other environments following previous *xoxF* assignations [78–80] (Supplementary Fig. S6). Further details on key features of the Anza-Borrego genomes, as unveiled by assessing the distribution of the 8668 groups of orthologous genes comprising the *Methylocaldum* pangenome, are described below (and in Supplementary Tables S4 and S5).

### *Methylocaldum* has four *pmoC* paralogs in addition to the canonical gene involved in methane oxidation

**Figure 5:**
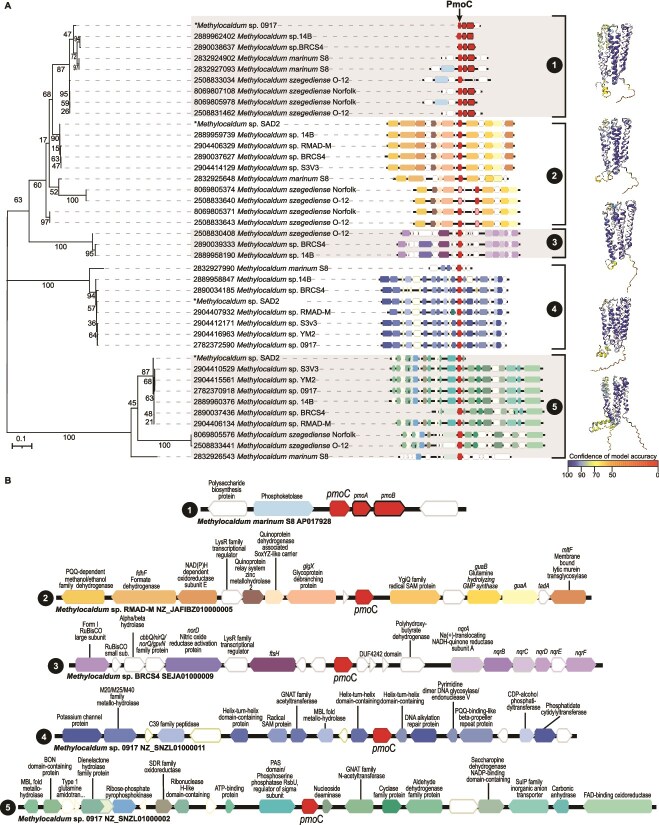
(A) Maximum likelihood tree representing the phylogenetic relationship of *Methylocaldum* particulate methane monooxygenase, based on the amino acid sequences of the *pmoC* gene. Next to each branch, the projection has a color-coded representation of the predicted functions for the genes in the vicinity of each *pmoC*. Next to each clade, brackets indicate with a number the type of *pmoC* corresponding to each clade. Type 1 PmoC is part of the canonical pMMO, whereas Types 2 to 5 correspond to the different stand-alone PmoC. Predicted AlphaFold models were based on the prediction obtained from the CryoEM structure of *Methylococcus capsulatus* (bath) pMMO in a native lipid nanodisc at 2.16 Angstrom resolution (ID 7S4J). Predicted structures are color-coded according to the confidence percentage in the model accuracy. (B) Gene function prediction for genes in the vicinity of each of the five types of *pmoC* from *Methylocaldum* strains. The canonical *pmoC* from 0917 was recovered from a metatranscriptome.

The *Methylocaldum* pangenome revealed the presence of several paralogs among the 39 *pmoC* retrieved (K10946), of which only nine formed parts of the canonical pMMO gene cluster (Fig. 5). The remaining stand-alone *pmoC* paralogs (that included neither *pmoA* nor *pmoB*) could be grouped into four distinctive gene contexts that were conserved even among representatives isolated from different continents (Fig. 5A and Supplementary Fig. S4). These stand-alone *pmoC* genes clades were named “Type 2 to 5” considering their conserved genomic context and phylogenetic placement to the canonical “Type 1” pMMO gene cluster (Fig. 5B and Supplementary Material). The immediate vicinity of the conserved genomic context of Type 2 *Methylocaldum pmoC* had genes involved with nucleoside modification or the biosynthesis of GMP and the generation of radical species by reductive cleavage of S-adenosylmethionine. The genes located near Type 3 *pmoC*s encode the degradation of polyhydroxybutyrate, an energy and carbon storage polymer [81], and the Na-translocating NADH-quinone reductase respiratory complex operon [82]. All Type 4 *pmoC* are located between two genes, one of which has a fumarate/nitrate reduction transcriptional regulator domain, and both contain helix-turn-helix domains. Thus, these genes likely have a role in either transcriptional regulation or DNA-binding processes [83], that in *Escherichia coli* have been linked to an oxygen-responsive transcriptional regulation to switch from aerobic to anaerobic metabolism [84]. The Type 5 *pmoC* clusters the *rsbU* gene, which has been shown to contribute to general stress sensing and response, as well as oxygen starvation [85, 86], and a putative nucleoside deaminase, followed by the GCN5-Related (GNAT-family) ***N***-Acetyltransferases, are known to contribute to a broad spectrum of cellular metabolic and regulatory functions [87].

To further investigate the stand-alone *pmoC* function, the in-silico simulations of the predicted tertiary structure of *Methylocaldum* PmoC types were carried out. The analyses showed a differential tertiary structure for all stand-alone PmoC, whereas still preserving their transmembrane domains (Supplementary Fig. S7 and Supplementary videos). Additionally, the search for the presence of the key amino acid residues forming the Cu D binding site, which recent structural studies had indicated as the methane oxidation catalytic center in PmoC [88] was conserved across all *Methylocaldum* PmoC types (Supplementary Fig. S8). Types 2 and 3 PmoCs differ from Type 1 only slightly in structure, suggesting that these stand-alone PmoC likely perform similar membrane anchoring and/or metabolite binding functions. Individual differences in residues compared to Type 1 might reflect lessened evolutionary pressure for folding outside of catalytic regions when the structure is not restricted by a multi-protein complex. Type 4 differs in the structure of the N-terminal signal peptide, suggesting that these proteins differ in their subcellular localization compared to the other PmoC types. Type 5 PmoC contains an ~40 residue region that extends the largest cytoplasmic domain. This additional catalytic region might reflect additional substrate specificities, substrate preferences, or new binding sites. Additional experimental work will be needed to confirm functional differences between these *pmoC* types.

Overall, the functional diversity of the genes in the immediate vicinity and the same orientation as the stand-alone *pmoC*, added to their differential tertiary structure, suggests that in *Methylocaldum* these paralogs are likely involved in other functions not related to CH_4_-oxidation (Fig. 5B and Supplementary Material).

### TRAP transporter system

A tripartite ATP-independent Periplasmic (TRAP)-type periplasmic transport system [89] was found exclusively in the Anza-Borrego *Methylocaldum* isolates and not in other *Methylocaldum* isolates (Fig. 4). TRAP transporters are one of the three known solute binding-protein-dependent systems which are characterized by their high-affinity for the uptake of substrates (the other two are the well-studied ATP-binding cassette and the more recently studied tripartite tricarboxylate transporters [90]). TRAP transporters do not require ATP hydrolysis and instead use transmembrane electrochemical gradients (usually sodium or other cations) to transport various molecules, including C4-dicarboxylates, sulfonate, and carboxyl-containing substrates [90, 91].

The putative TRAP-type transport system in the Anza-Borrego *Methylocaldum* isolates was found to be encoded by three genes—a TRAP transporter component, a periplasmic component, and the fused version of the large and small permease component. The putative TRAP-type gene cluster in the Anza-Borrego *Methylocaldum* isolates resembles an evolved variant of the TRAP transporters from *Treponema pallidum* [90, 92], which has been predicted to transport hydrophobic nutrients through the periplasm [93–95]. The involvement of this system in transporting substrates in response to reduced water availability has been suggested in a metaproteomic study reporting that the TRAP-type protein abundances produced by populations of Pseudomonadota (from the genus *Acidithrix, Aureimonas, Niastella*, and *Pedobacter*) and Actinomycetota (from the genus *Jiangella*) were higher in soils subjected to a regulated irrigation-deficit [96]*.* Considering that the methanotrophic bacteria have very limited ability to utilize extracellular organic carbon, the role of the TRAP transport mechanisms in *Methylocaldum* deserves a thorough investigation.

### Interaction among methanotrophic microbiome members

**Figure 6:**
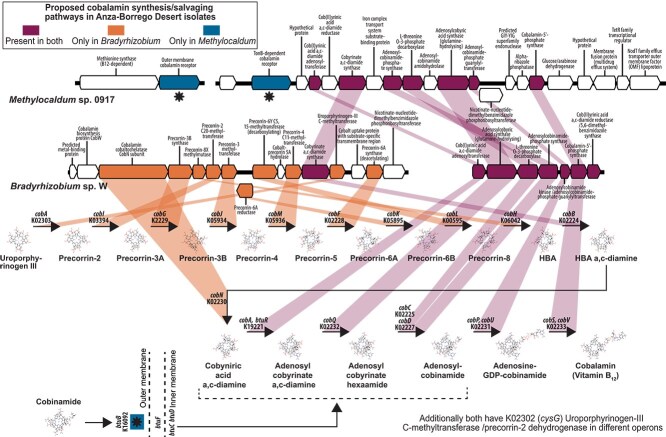
Genomic potential for cobalamin (vitamin B_12_) de novo synthesis and salvaging by Anza-Borrego isolates of *Bradyrhizobium* and *Methylocaldum,* respectively. The required genes for de novo cobalamin synthesis are present in the *Bradyrhizobium* genome. The absence of the initial required genes for de novo synthesis and the presence of two paralogs of the cobalamin outer membrane cobalamin receptor and transporter gene, *btu*B, indicates that *Methylocaldum* relies on a salvaging pathway of cobalamin.

*Methylocaldum* strains were co-isolated with *Bradyrhizobium,* and the challenges for their separation suggested some level of dependency for the methanotrophs. A genomic comparison revealed that *Methylocaldum* possesses only the genes necessary for salvaging cobalamin (vitamin B_12_). In contrast, *Bradyrhizobium* has all the necessary genes for the de novo synthesis of the essential cofactor (Fig. 6). *Methylocaldum* has two methionine synthesis pathways, dependent and independent of vitamin B_12_. However, considering that the association with *Bradyrhizobium* is advantageous for *Methylocaldum* growth (data not shown), we speculated that the B_12_ exchange supports the symbiotic interactions between the species. It has been previously demonstrated that rhizobia can stimulate the growth of methanotrophs via excreted cobalamin [97]. Our finding provides genetic evidence for such dependencies. It should be mentioned that the B_12_ exchange is perhaps the most common interaction between microbes in complex soil or aquatic communities. Several carbon and nitrogen catabolism pathways can also rely on cobalamin [98], and metagenomic studies indicate that only <10% of soil prokaryotes encode the genetic potential for de novo synthesis [99].

Both rhizobia and methanotrophs have been shown as key microbial partners for N_2_ fixation in non-leguminous plants [100]. Even though expected for *Bradyrhizobium*, additionally, all Anza-Borrego *Methylocaldum* strains have the genetic potential for dinitrogen (N_2_) fixation, including *nifH* (K02588) and *nifK* (K02591), in addition to *nifB* (K02585), *nifD* (K02586), *nifQ* (K15790), *nifU* (K04488), and *nifZ* (K02597) (Supplementary Fig. S9). Therefore, *Methylocaldum* has the enzymatic inventory to fix nitrogen, providing an advantage for both bacteria and its host plant in the low-nutrient desert environment.

**Figure 7:**
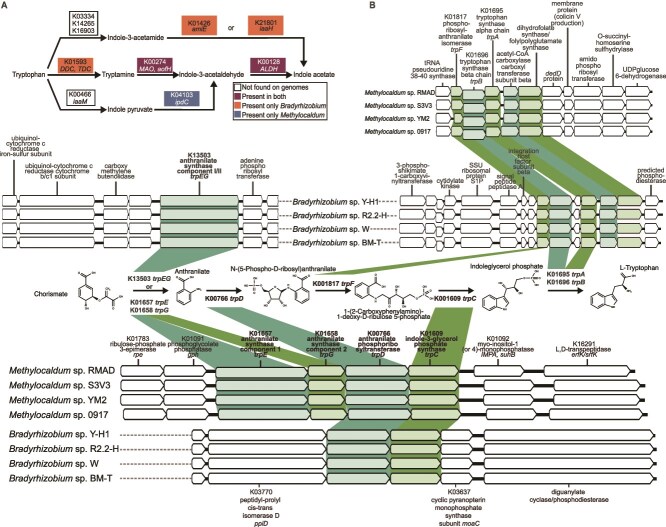
Genomic potential for tryptophan and indole-3-acetic acid (IAA) synthesis in Anza-Borrego isolates of *Bradyrhizobium* and *Methylocaldum.* (A) Comparison of metabolic potential for the synthesis of IAA by Anza-Borrego isolates. (B) The major difference in their tryptophan synthesis pathway relies on the gene encoding the initial step enzymes; *Bradyrhizobium* has the fused version of the anthranilate synthase TrpEG, and *Methylocaldum* has its two components separately.

Searches for metabolisms favoring the interaction of the *Methylocaldum-Bradyrhizobium* consortia with plants pointed to the different pathways for tryptophan synthesis and its subsequent metabolization to indole-3-acetic acid (IAA) production as relevant. *Methylocaldum* and *Bradyrhizobium* both have the necessary genomic potential for tryptophan synthesis (Fig. 7). IAA is an important auxin in plants, which acts as a phytohormone regulating plant growth and also mediates bacterial physiology [101]. *Methylocaldum* and *Bradyrhizobium* have the complete gene set necessary for IAA production via tryptamine and indole-3-acetaldehyde. Moreover, *Bradyrhizobium* can also potentially produce IAA via indole-3-acetamide, whereas *Methylocaldum* could also produce it via indole pyruvate (Fig. 7B). Production of IAA had been found in a majority of plant-interacting bacteria and had been shown to confer benefits to the host plant, and also an advantage under environmental stress for bacteria [101].

## Conclusions

This study was inspired by a high methane sink observed in arid ecosystems via *in situ* measurements almost three decades ago [19] and a more recent remote-sensor-based demonstration of arid ecosystems as “black holes” of methane, i.e., environments with methane levels significantly below the average [102]. Here, we aimed to better understand the underlying biological means of arid methane sinks. We investigated methane cycling in the Anza-Borrego State Park, starting with enrichments in 2016, followed by additional sampling sets in 2018, 2020, and 2023. Each time, we isolated microbes from vegetated sites, which inspired a deeper investigation of methanotrophic biome structure via metagenomic studies and *in situ* methane flux measurements. Integrated, the data highlights the importance of interaction among arid biomes, especially soil microbes and arid vegetation, for atmospheric methane consumption. Plant-supported methane flux shows daily dynamics, suggesting yet-to-be-determined links with the plant’s photosynthetic activity. This observation became the foundation for the reverse chimney hypothesis proposed here (Fig. 1D), which could partially explain why deserts constitute natural CH_4_ sinks. This hypothesis was coined as the opposite of the chimney effect [70–73], in which plants transport CH_4_ from the anoxic soil layers from their roots through their vascular system to their leaves and release it into the atmosphere (Fig. 1C). The reverse chimney hypothesis proposes that in dryland ecosystems (i.e., low organic matter and limited water availability) plants transport CH_4_ and oxygen from the atmosphere through their vascular system down to the soil, where it is consumed and converted by methanotrophic microbiota (Fig. 1D). It is tempting to speculate that plants accelerate desert methane sinks by providing unique ecosystem support for methanotrophic microbes. Research on how plants contribute to the modulation of CH_4_ fluxes has only recently started to be explored. The results presented here encourage future studies aimed at understanding how the CH_4_ concentration, macronutrients (i.e., N, S, or P), and moisture levels of the soil determine whether a system functions as a sink or source of CH_4_. The plant-promoting properties of the desert *Methylocaldum* species will be described in a separate study.

The *Methylocaldum* clade is the most prominent methanotroph in the studied ecosystem, and is the most likely contributes to the observed atmospheric methane consumption. Up to date, none of the *Methylocaldum* spp. were reported as high-affinity methane oxidizers [103, 104]. Thus, their role in the soil methane cycling needs to be re-evaluated. Genomic analysis of *Methylocaldum* isolates provides insights into the unique metabolic features of the dominant methanotroph in Anza-Borrego soil, contributing to the understanding of microbial adaptation in arid environments. These adaptations include the presence of multiple stand-alone *pmoC* paralogs in *Methylocaldum*, which may have functions beyond methane oxidation or contribute to high-affinity methane oxidation. The TRAP found in Anza-Borrego *Methylocaldum* isolates might play an important role in supporting substrate transport under reduced water availability.

Genomic evidence suggests that *Methylocaldum* may rely on a symbiotic relationship with *Bradyrhizobium* for cobalamin (vitamin B_12_) due to its lack of de novo synthesis genes. Additionally, both *Methylocaldum* and *Bradyrhizobium* possess the necessary genes for tryptophan synthesis and IAA production, indicating a potential interaction that benefits plant growth and bacterial adaptation in arid environments. Establishing beneficial rhizosphere microbiomes in drylands could enhance soil stability by producing an extracellular matrix [105], promoting vascular plant growth, or generating a positive feedback loop that supports the proposed reverse chimney effect.

These findings are the starting point for further research on how CH_4_ concentrations, macronutrients, and soil moisture levels influence the role of plant-microbiome biomes in modulating CH_4_ fluxes at different scales across seasons in arid ecosystems. Further research is needed to explore the impact of different vegetation types, environmental conditions, and soil microbiomes on terrestrial CH_4_ fluxes.

## Supplementary Material

PmoC_Methylocaldum_Type_1_wraf026

PmoC_Methylocaldum_Type_2_wraf026

PmoC_Methylocaldum_Type_3_wraf026

PmoC_Methylocaldum_Type_4_wraf026

PmoC_Methylocaldum_Type_5_wraf026

Supplementary_Material_Revision_ISMEJ-D-24-01549R2_(1)_wraf026

## Data Availability

The datasets generated and analysed during the current study are available in the JGI-IMG/MER repository, https://img.jgi.doe.gov/mer/.
