# Supplementary material for: Atmospheric methane consumption in arid ecosystems acts as a reverse chimney and is accelerated by plant-methanotroph biomes
Source: ISME J. 2025 Mar 2;19(1):wraf026. doi: 10.1093/ismejo/wraf026 (PMC11931723; doi:10.1093/ismejo/wraf026)
Supplement: Supplementary_Material_Revision_ISMEJ-D-24-01549R2_(1)_wraf026 [file supplementary_material_revision_ismej-d-24-01549r2_(1)_wraf026.docx]

**Supplementary Information**

**Title: Atmospheric methane consumption in arid ecosystems acts as a reverse chimney and is accelerated by plant-methanotroph biomes**

Nathalie A. Delherbe^1^, Oscar Gomez^1^, Alvaro M. Plominsky^2^, Aaron Oliver^2^, Maximino Manzanera^3^, and Marina G. Kalyuzhnaya^1#^

^1^ Department of Biology, San Diego State University, San Diego, CA 92129, USA

^2^ Marine Biology Research Division, Scripps Institution of Oceanography, University of California San Diego, La Jolla, CA, 92037, USA

^3^ Institute for Water Research and Department of Microbiology, University of Granada, 18071 Granada, Spain

# Corresponding author: mkalyuzhnaya@sdsu.edu

**Materials and Methods**

**Enrichment Cultures**

Methanotrophic cultures were isolated from soil samples collected in Spring 2015 and 2016, using an enrichment strategy described in [1] and were cultivated using P_0%_ medium [2], consisting of 1 g L^-1^ of KNO_3_, 0.2 g L^-1^ of MgSO_4_ x 7H_2_O, 0.02 g L^-1^ of CaCl_2_ x 2H_2_O and 1 ml of 1000x trace solution. After autoclaving (at 121°C for 45 minutes), a sterile phosphate solution (1/20 v/v), containing 5.44 g L^-1^ of KH_2_PO_4_ and 5.68 g L^-1^ Na_2_HPO_4_ (pH of 6.8), was added. Trace solution (1000x) consisted of 5 g L^-1^ of Na_2_EDTA, 2g L^-1^ of FeSO_4_ x 7 H_2_O, 0.3g L^-1^ of ZnSO_4_ x7 H_2_O, 0.03 g L^-1^ of MnCl_2_ x 4 H_2_O, 0.2 g L^-1^ of CoCl_2_ x 6 H_2_O, 1.2 g L^-1^ of CuSO_4_ x 5 H_2_O, 0.3 g L^-1^ of Na_2_O_4_W x 2H_2_O, 0.05 g L^-1^ of NiCl_2_ x 6 H_2_O, 0.05 g L^-1^ of Na_2_MoO_4_ x 2 H_2_O, and 0.03 g L^-1^ of H_3_BO_3_.

A subset of soil samples collected on May 30^th^, 2016, were used for culture enrichment. Soil (1 to 2 g) was added to 25 ml of 1:3 diluted P0_%_ media in 250 ml borosilicate bottles with rubber septa, and supplemented with 1 ml of 100% methane. After 24 h, 5 ml of methane were added, and samples were incubated for 3-4 days at 30^o^C with agitation (200 rpm). Cultures exhibiting increased turbidity were transferred into 50 ml P_0%_ media in 250 ml bottles and supplemented with pressurized 50 ml of methane (20%). For each successful enrichment (i.e., culture turbidity increased after each passage at least three passages), serial dilutions were performed and then spread-plated on solid P_0%_ media to produce isolated colonies.

**Pure culture isolation via co-plating method**

Individual clones were selected and re-plated. However, in many cases, only a stable co-culture was obtained. The separation of methanotrophic isolates from the consortia was only achieved by the co-plating technique. Briefly, a petri dish was separated into two zones; co-culture was plated on one side and incubated for 1-2 days. Then, single colonies were streaked on the second half of each plate and incubated for an additional 4-5 days. This method helped separate pure methanotrophic cultures from stable co-cultures. Isolation of heterotrophic satellite cultures was carried out by plating on P_M_ and/or P_NB_ media.

**DNA preparation, sequencing, assembly, and annotation for metagenomics.**

Samples collected in 2023 were extracted with a phosphate buffer and high pH lysis buffer protocol adapted from Povedano-Prieto et al.[3] and Zeugin and Hartley[4] , aiming to recover DNA from high-sand content samples adsorbing DNA [5]. Briefly, 0.25 g of each sample was placed on 2 ml screw-cap tube with DNA-free 3mm glass beads and 400 μL of Na2HPO4 (120 mM pH 8.5), plus 600 μl of lysis buffer (100 mM Tris-HCl [pH 9.5]; 100 mM EDTA [pH 8.5]; 100 mM NaCl), 20 ul SDS 10%, 24 μl freshly thawed lysozyme (10 mg/ml) and 2 μl proteinase K (20 mg/ml). Cells were mechanically broken by bead beating on a vortexGenie2 (Scientific Industries) at maximum speed for 40 min in a cold room (4°C). The supernatant was processed through an organic extraction and isopropanol precipitation [4] [5]. to obtain the DNA. The integrity of the DNA was verified by 1% agarose gel electrophoresis and quantified on a Qubit utilizing a high-sensitivity kit.

***Methylocaldum* phylogeny**

The 16S rRNA gene and formate dehydrogenase gene phylogeny of the new and previously characterized *Methylocladum* strains were reconstructed as previously described [6]. Maximum likelihood trees for theRuBisCO were generated based on the amino acid sequences of the large subunit gene (*rbc*L) from representative species of the three domains of life. The model calculated to have the lowest BIC scores for this set of sequences corresponded to LG+G+I.

**RESULTS AND DISCUSSION**

**Anza-Borrego Soil Metagenomes:** The number of reads obtained for each 2023 metagenome are shown in the following table.

**
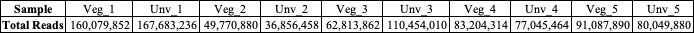
**

**Formaldehyde oxidation:** All the analyzed genomes have the potential for C_1_ interconversion from tetrahydrofolate (THF) to 10-formyl-THF, but only present a pair of genes for the reductive acetyl-CoA pathway (i.e., the methylene-THF reductase and formate-THF ligase) which is required for the complete THF methylene tetrahydrofolate pathway (Supplementary Table 4). Although all *Methylocaldum* genomes lacked the genes to synthesize tetra-hydromethanopterin (H4MPT), they present genes involved on the reversible transformation of formylmethanofuran to 5-Formyl-5,6,7,8-H4MPT (methenyl-H4MPT cyclohydrolase, formylmethanofuran dehydrogenase, and formyl-methanofuran-H4MPT N-formyltransferase). Therefore, potentially having some capacity to use methanopterin to transfer C_1_ fragments between formyl and methyl groups.

**Formate:** Formate can be transformed to CO_2_ and NADH by the enzyme formate dehydrogenase (FDH). Interestingly, all the *Methylocaldum* genomes have three copies of this gene, but strains O-12 and S8 have only one and two copies, respectively. A previously generated phylogenetic analysis revealed that each one of the copies found in the *M. gracile* group corresponded to the different types of this enzyme reported in *Methylorubrum extorquens* AM1: *fdh1A*, *fdh2A*, and *fdh3A*. The *fdh* genes of strains S8 and O-12 formed a sister group to the *fdh*3*A* cluster [6].

**Carbon assimilation: ribulose monophosphate pathway.** All the genomes analyzed had the potential for the assimilation of formaldehyde via ribulose monophosphate (RuMP) pathway. Proposed as one of the most efficient formaldehyde assimilation routes (considering ATP consumption and biomass yield) [7]. All *Methylocaldum* genomes have two copies for the 3-hexulose-6-phosphate synthase gene (K08093), but each genome has only one copy for the 6-phospho-3-hexuloisomerase (K08094).

**Carbon assimilation: Serine pathway.** Formaldehyde can alternatively be assimilated through the serine pathway after its transformation to methylene-THF [8]. Although they had some of the components, such as: one copy for serine-glyoxylate transaminase (K00830) in all genomes, the hydroxypyruvate reductase (K12972) found only on *Methylocaldum* sp. SAD2, and one copy for D-3-phosphoglycerate dehydrogenase (K00058) found in all genomes.

**Carbon assimilation: Calvin cycle**. Previously, the presence of the key enzyme ribulose 1,5-bisphosphate carboxylase/oxygenase (RuBisCo) on some methanotrophs suggested the possibility of CO_2_ fixation through the Calvin–Benson–Bassham (CBB) pathway in these microorganisms [8]. A single copy for each subunit of the Ribulose 1,5-bisphosphate carboxylase, (RuBisCO-like protein), large subunit *rbcL* (K01601), and small subunit *rbcS* (K01602) were found for each *Methylocaldum* genome. Thus, further supporting the potential for members of this genus to utilize CO_2_ as carbon source through the CBB cycle, as it has been shown in *Methylococcus capsulatus* Bath [9]. A phylogenetic analysis revealed that the large subunit *rbcL* of the Anza-Borrego *Methylocaldum* strains form a sub-clade within the Type I RuBisCO found in *Pseudomonadota* (*Proteobacteria*), Cyanobacteria, and higher plants (Supplementary Figure 6). This suggests an evolutionary history of the RcBL of *Methylocaldum* and *Methylococcus*, related to Type I-A and I-B rubisco forms, which first appeared under the primitive low O_2_ atmospheric conditions, and developed alternative strategies to compensate the increasing concentrations of O_2_ of the post-Great Oxidation Event through carbon concentrating mechanisms (CCM), such as carboxysomes in cyanobacteria [10] and pyrenoid-based in *Chlamydomonas* [11], even though similar structures had been only described on *Verrucomicrobiota* (*Verrucomicrobia*) members [12].

**Nitrogen metabolism, nitrogen fixation.** All the analyzed strains have the genetic potential for dinitrogen (N_2_) fixation, including *nifH* (K02588) and *nifK* (K02591), in addition to *nifB* (K02585), *nifD* (K02586), *nifQ* (K15790), *nifU* (K04488), and *nifZ* (K02597). Additionally, all *Methylocaldum* isolate genomes in this study have a single copy for the two-component system, NtrC family, nitrogen regulation sensor histidine kinase NtrY (K13598) (Supplementary Table 3).

**Anza-Borrego *Methylocaldum* has additional *xoxF*-type methanol dehydrogenases.** All ten *Methylocaldum* genomes had a single copy for each subunit (*mdh1* and *mdh2*) of the pyrroloquinoline-quinone-dependent methanol dehydrogenase (MDH). This enzyme is unique to methanotrophic/methylotrophic bacteria and donates two electrons to cytochrome c_L_ to produce formaldehyde [13]. Previously, our group described that in addition to MDH, the genomes of *Methylocaldum marinum* S8, *Methylocaldum szegediense* O-12, and *Methylocaldum szegediense* Norfolk present the lanthanide-dependent *xoxF*-type methanol dehydrogenase types *xoxF3*, and *xoxF5* [6]. Notably, all the Anza-Borrego *Methylocaldum* isolates from this study presented additional *xoxF3*-type methanol dehydrogenase paralogs (Supplementary Figure S6). Moreover, one of the xox*F3* paralogs formed a sister group with only Anza-Borrego genes (Supplementary Figure S6). Since each *xoxF* clade has been reported to have variable catalytic efficiencies [14], this might also be true for the various *Methylocaldum’s xoxF3* subtypes.

**Novel stand-alone *pmoC* reveal a conserved functional diversity among *Methylocaldum* genomes.** While the canonical *pmoC, pmoA* and *pmoB* genes comprising the particulate methane monooxygenase were denoted here as the ‘Type 1’ *pmo*-cluster, an additional 4 stand-alone *pmoC* genes that had neither *pmoA* nor *pmoB* in their genomic neighborhood were found (Fig. 5A). These stand-alone *pmoC* genes were named ‘Type 2 to 5’ considering their genomic context and phylogenetic placement (Fig. 5A).

The immediate vicinity of the genomic context conserved for Type 2 *pmoC* included the presence of radical S-adenosylmethionine (SAM) protein YgiQ, as well as genes coding for a guanosine monophosphate (GMP) synthesis*, guaA* and *guaB*, followed by a tRNA (adenine-34) deaminase. The YbiQ enzyme has been reported to generate radical species by reductive cleavage of SAM through an unusual Fe-S center, a two-step process that produces/consumes NADH and consumes/produces ATP [15]. Cobalamin-dependent radical SAM enzymes with glutamine C-methyltransferase activity have been reported for some methanotrophs [16]. More information is available for the archaeal radical SAM protein, which has been reported to synthesize a structurally complex modified nucleoside called archaeosine [17]. This archaeosine is similar to the commonly found queuosine, a structurally complex, non‐canonical RNA nucleoside present in bacteria, part of the anticodon loop of certain tRNA [18]. Modified tRNAs have been shown to play a crucial role in offering stable tRNA structures at high temperatures in the extremely-thermophilic bacterium *Thermus thermophilus* [19]. Moreover, sequence similarity network analysis indicated that most available radical SAM sequences belong to bacteria, followed by archaea [20]. Interestingly, comparatively more predicted radical SAM proteins had been found on methanogenic archaea genomes than in other archaea and bacteria; however, the function of half of them remains elusive [20, 21].

Type 3 *pmoC* stand-alone genes were only found in strains O-12, BRCS4 and 14B. The immediate vicinity of its conserved genomic context has two small hypothetical proteins, polyhydroxy butyrate depolymerase, which catalyzes the degradation of the biopolymer polyhydroxybutyrate (PHB) utilized as nutrient storage [22], and the *nqr* Na-translocating NADH-quinone reductase operon, which is a respiratory complex that couples the oxidation of NADH to the translocation of sodium ions across the membrane [23]. Suggesting a role of PmoC type 3 in balancing catabolic and anabolic functions.

The immediate vicinity of the Type 4 *pmoC* has genes with a RsbU-like domain with a fused phosphatase and signaling PAS-domain module. PAS-domain S-box proteins are crucial in signal transduction mechanisms in bacteria, and a known sensor domain for complex behavioral pathways in bacteria including motility and quorum sensing [24]. The sigma-B regulation protein RsbU is involved in regulating the general stress-response in bacteria [25]. The PAS-domain within RsbU has a reported role in sensing stresses (i.e., carbon, phosphate, or oxygen starvation) [26], and have been implicated in sensing redox potential changes [27]. This suggests a possible role for *pmoC* type 4 in sensing or responding to environmental cues/stressors.

Type 5 *pmoC* is in between two helix-turn-helix domains, suggesting either a role in transcriptional regulation, DNA binding processes, or being the catalytic domain of another enzyme of a yet unknown function [28]. The helix-turn-helix behind *pmoC* has a fumarate/nitrate reduction transcriptional regulator Fnr domain, which in *Escherichia coli* is an oxygen-responsive transcriptional regulator, necessary for switching from aerobic to anaerobic metabolism [29]. Additionally, the Type 5 *pmoC* conserved genomic context, contains a DNA alkylation repair protein, for repairing damage by alkylating agents [30]. Followed by a pyrimidine dimer DNA glycosylase/endonuclease V, essential for repairing UV-induced pyrimidine dimers (DNA damage) [31].

**Supplementary Table S1.** Pure methanotrophic and satellite cultures obtained in this study

| **Metagenome ID** | **Sequencing**  **Center** | **IMG Genome**  **ID** | **Genome Size (assembled)** | **Gene Count**  **(assembled)** |
| --- | --- | --- | --- | --- |
| S1+v_20-13C | DOE JGI | 3300020131 | 187019236 | 409296 |
| S3+v_10-13C | DOE JGI | 3300020146 | 317105193 | 650436 |
| S3+v_5-13C | DOE JGI | 3300020147 | 312907534 | 653428 |
| S1+v_10-13C | DOE JGI | 3300020152 | 269467853 | 523210 |
| S1_20 | DOE JGI | 3300020154 | 415175613 | 915500 |
| S1+v_5-13C | DOE JGI | 3300020156 | 287312560 | 512354 |
| S1+v_10 | DOE JGI | 3300020181 | 882401169 | 2245240 |
| S1_10 | DOE JGI | 3300020202 | 1340989938 | 3214547 |
| S1_5 | DOE JGI | 3300020215 | 1224300329 | 2884978 |
| S3_20-13C | DOE JGI | 3300021057 | 204709815 | 476996 |
| S1_5-13C | DOE JGI | 3300021061 | 266934236 | 639860 |
| S1_10-13C | DOE JGI | 3300021062 | 213311580 | 526868 |
| S3_10-13C | DOE JGI | 3300021066 | 227159040 | 579838 |
| S3+v_20-13C | DOE JGI | 3300021067 | 278253160 | 604522 |
| S1+v_20 | DOE JGI | 3300021184 | 596000187 | 1723296 |
| S3+v_5 | DOE JGI | 3300024426 | 800577437 | 2127945 |
| S3+v_20 | DOE JGI | 3300024430 | 705051987 | 1739013 |
| ABUNV3_2023 | UC Davis | 3300069200 | 1267035382 | 3012337 |
| ABUNV2_2023 | UC Davis | 3300069201 | 348486353 | 931977 |
| ABUNV1_2023 | UC Davis | 3300069202 | 1461045267 | 3838297 |
| ABUNV4_2023 | UC Davis | 3300069302 | 812910625 | 2094070 |
| ABUNV5_2023 | UC Davis | 3300069303 | 562751058 | 1462859 |
| ABVEG1_2023 | UC Davis | 3300069304 | 1243370612 | 3241385 |
| ABVEG2_2023 | UC Davis | 3300069305 | 409272842 | 1141261 |
| ABVEG3_2023 | UC Davis | 3300069306 | 623143531 | 1677139 |
| ABVEG4_2023 | UC Davis | 3300069307 | 914596593 | 2333217 |
| ABVEG5_2023 | UC Davis | 3300069308 | 1036166019 | 2560090 |

**Supplementary Table S2.** Pure methanotrophic and satellite cultures obtained in this study

| **Culture** | **IMG Genome**  **ID** | **Soil sample (Year collected)** | **Description** | **Plant (soil or material)** |
| --- | --- | --- | --- | --- |
| *Methylocaldum* sp. 0917 | 2781126057 | ABDSP (2015) | Methanotroph, axenic culture | Brittlebush (*Encelia farinose)* Rhizosphere 6-10 cm |
| *Methylocaldum* sp. S3V3 | 2904410029 | ABDSP (2016) | Methanotroph, axenic culture | Desert Willow *Chilopsis linearis*, Rhizosphere 5cm deep |
| *Methylocaldum* RMAD-M | 2904404936 | ABDSP (2017) | Methanotroph, axenic culture | Brittlebush (*Encelia farinose)* root |
| *Mehylocaldum* YM2 | 2904414868 | ABDSP (2017) | Methanotroph, axenic culture | Brittlebush (*Encelia farinose)* flower |
| *Methylosinus* sp. SAV-B | N/A | ABDSP (2015) | Methanotroph, axenic culture | Brittlebush (*Encelia farinose)* Rhizosphere 6-10 cm |
| *Methylosinus* sp. SAV-2 | 2784132047 | ABDSP (2015) | Methanotroph, axenic culture | Desert Willow *Chilopsis linearis*, Rhizosphere 5cm deep |
| *Bradyrhizobium* sp. W | 2784132048 | ABDSP (2015) | Satellite culture | Brittlebush (*Encelia farinose)* Rhizosphere 6-10 cm |
| *Bradyrhizobium* sp. R2.2-H | 2781126058 | ABDSP (2017) | Satellite culture | Brittlebush (*Encelia farinose)* root |
| *Bradyrhizobium* sp. BM-T | 2784132071 | ABDSP (2017) | Satellite culture |  |
| *Bradyrhizobium* sp. Y-H1 | 2781126056 | ABDSP (2017) | Satellite culture | Desert Sunflower (*Geraea canescens)* |
| *Neorhizobium* sp. R1B | 2784132052 | ABDSP (2017) | Satellite culture | Desert Sunflower (*Geraea canescens)* |
| *Neorhizobium* sp. S3V5DH | 2784132046 | ABDSP (2016) | Satellite culture | Desert Willow *Chilopsis linearis*, Rhizosphere 5cm deep |
| *Caulobacter* sp. H1 | 2824593709 | ABDSP (2016) | Satellite culture | NR |
| *Hansschlegelia* sp. R3 | N/A | ABDSP (2017) | Satellite culture | Desert Sunflower (*Geraea canescens) roots* |
| *Methylobacterium* sp. R2-1 | 2824205890 | ABDSP (2017) | Satellite culture | Brittlebush (*Encelia farinose)* root |
| *Sphingopyxis* sp. NOR-H1-2 | N/A | ABDSP (2017) | Satellite culture | *Nerium oleander* |

NR: plant species was not recorded or identified

**Supplementary Table S3.** An overview of *Methylocaldum* strains is included in this study. Type strains are highlighted in bold.

| Genus/Specie | Strain | Physiological Highlights | Temp. range (°C) | C1 utilization | Isolation Source/ Country | GC (%) | Geno-me Length (Mb) | Genome NCBI accession or /and IMG ID | Gene sequence available | | | Reference |
| --- | --- | --- | --- | --- | --- | --- | --- | --- | --- | --- | --- | --- |
|  |  |  |  |  |  |  |  |  | 16S | *mmo*X | *pqq xox mxa* |  |
| *Methylocaldum* sp. | 0917 |  |  |  | Soil-sand / USA, Anza Borrego Desert, CA | 57.97 | 5.19 | 2781126057 | yes | IMG | IMG | This study |
| *Methylocaldum* sp. | YM2 |  |  |  | Soil-sand / USA, Anza Borrego Desert, CA | 57.97 | 5.19 | 2904414868 | yes | IMG | IMG | This study |
| *Methylocaldum* sp. | S3V3 |  |  |  | Soil / Anza-Borrego Desert, CA | 57.98 | 5.19 | 2904410029 | yes | IMG | IMG | This study |
| *Methylocaldum* sp. | RMAD-M |  |  |  | Soil-sand / USA, Anza Borrego Desert, CA | 58.07 | 5.45 | SAMN17693797 / 2904404936 | yes | IMG | IMG | This study |
| ***Methylocaldum* *gracile **** | NCIMB:11912, VKM: **14L ^T^** | (Moderate) Thermophilic | 20 to 47 |  | Fresh water mud, Active sludge / Russia | n/a | n/a | n/a | yes | NCBI | no | [32-34] |
| ***Methylocaldum* *marinum **** | personal:**S8 ^T^**, NBRC: 109686, DSM:27392 | Thermo-tolerant, Marine | 20 to 47 | Methane,methanol | Marine sediment / Japan, Kagoshima Bay | 58.7 | 6.09 | NZ_AP017928.1 / 2832923104 | yes | IMG | IMG | [34, 35] |
| ***Methylocaldum szegediense **** | **OR2 ^T^** | Moderate thermophilic | 37 to 62 | Methane | Underground hot spring connected to a natural gas field near Szeged / Hungary | n/a | n/a | n/a | yes | yes | no | [32, 34, 36] |
| ***Methylocaldum tepidum **** | **LK6^T^** | Thermo-tolerant | 30 to 47 | Methane, methanolformate, or MA* | Agricultural soil from Rothamsted Experimental Station / England (Harpenden, Hertfordshire) | n/a | n/a | n/a | yes | NCBI |  | [32, 35] |
| *Methylocaldum szegediense* | O-12 | Moderate thermophilic, Obligate Methanotroph | 30 to 61 | Methane | Manure /Russia Pushchino Moscow | 57.1 | 5.02 | NZ_ATXX00000000.1 / 2508501066 | yes | IMG | IMG | [34, 37] |
| *Methylocaldum* sp. | 14B | methane oxidation in the presence of H2S, fast growth | 37 to 42 | Methane | Solid state anaerobic digester fed corn stover / USA, Wooster, OH | 58.2 | 5.62 | SAMN05933571 / 2889957184 | yes | IMG | IMG | [38-41] |
| *Methylocaldum* sp. | BRCS4 |  |  |  | Cow manure / United Kingdom | 58.1 | 5.9 | SAMN10845862 / 2890033825 |  | IMG | IMG |  |
| *Methylocaldum* sp. | SAD2 | methane oxidation in the presence of H2S | 30 to 37 |  | Hydrogen sulfide-rich anaerobic digester / USA, Wooster, OH | 58.2 | 5.91 | SAMN05933458 |  |  |  | [38, 41, 42] |
| *Methylocaldum* sp. | 0510-P-2 | resilience to a high level of pyrene |  |  | Landfill upland soil / Taiwan landfill had been completed for 10 years and has planted trees | n/a | n/a | n/a | yes | NCBI |  | [43, 44] |
| *Methylocaldum* sp. | 05J-I-7 | resilience to a high level of pyrene |  |  | Landfill upland soil / Taiwan landfill had been completed for 10 years and has planted trees | n/a | n/a | n/a | yes | NCBI |  | [43-45] |
| *Methylocaldum* sp. | 5FB |  |  |  | Soil / Germany, Hünfeld, Rendzina (Leptosol), Mixed forest (Fagus sylvatica, Quercus sp., scattered Pinus sylvestris) | n/a | n/a | n/a | yes | yes | yes | [46] |
| *Methylocaldum* sp. | BFH1 | Moderately thermophilic and acid-tolerant obligate methanotrophs | 30 to 60 | Methan,methanol | Soil, Tropical topsoil (5 cm depth) / Bangladesh, small cave heated by a flame of natural methane gas leakage or surface gas blowout | n/a | n/a | n/a | yes | no | no | [45] |
| *Methylocaldum* sp. | dr65 |  |  |  | landfill cover soil | n/a | n/a | n/a | yes | no | no |  |
| *Methylocaldum* sp. | E10_A |  |  |  | Soil / Germany, Eiterfeld , Parabraunerde (Luvisol), Farmland (wintergrain) | n/a | n/a | n/a | yes | yes | yes | [47] |
| *Methylocaldum* sp. | H-11 | Moderately thermophilic, Obligate Methanotroph | 30 to 61 | Methane Only | Silage / Russia Pushchino Moscow | n/a | n/a | n/a | yes | no | no | [34, 37, 45] |
| *Methylocaldum* sp. | r6f | Thermophilic |  |  | Landfill cover soil | n/a | n/a | n/a | yes | no | no |  |

*MA, methylamine

**Supplementary Table S4:** Nitrogen metabolism key genes presence in *Methylocaldum* strains

|  |  |  |  |  | Assimilatory nitrate reduction (1st step only) | Denitrification | | | | Nitrification |  | | | Dissimilatory nitrate reduction (2n step only) | | | Nitrogen fixation | | | |
| --- | --- | --- | --- | --- | --- | --- | --- | --- | --- | --- | --- | --- | --- | --- | --- | --- | --- | --- | --- | --- |
|  | ***nrt*** | ***nrtA*** | ***nrtB*** | ***nrtC*** | ***nasA*** | ***nirK*** | ***norC*** | ***norB*** | ***hao*** | | | ***hcp*** | ***nirB*** | | ***nirD*** | ***nifD*** | | ***nifH*** | ***nifK*** |  |
| **KO ID** | K02575 | K15576 | k15577 | k15578 | K00372 | K00368 | K02305 | K04561 | K10535 | | | K05601 | K00362 | | K00363 | K02586 | | K02588 | K02591 |  |
| ***Methylocaldum* sp. 0917** | + | + | + | + | + | - | + | + | + | | | + | + | | + | + | | + | + |  |
| ***Methylocaldum szegediense* O-12** | + | - | - | - | + | + | + | + | + | | | + | + | | + | + | | + | + |  |
| ***Methylocaldum* sp. RMAD-M** | + | + | + | + | + | - | + | + | + | | | + | + | | + | + | | + | + |  |
| ***Methylocaldum* sp. BRCS4** | + | + | + | + | + | - | + | + | + | | | + | + | | + | + | | + | + |  |
| ***Methylocaldum* sp. YM2** | + | + | + | + | + | - | + | + | + | | | + | + | | + | + | | + | + |  |
| ***Methylocaldum marinum* S8** | + | + | + | + | + | - | + | + | + | | | + | + | | + | + | | + | + |  |
| ***Methylocaldum* sp. S3v3** | + | + | + | + | + | - | + | + | + | | | + | + | | + | + | | + | + |  |
| ***Methylocaldum* sp. 14B** | + | + | + | + | + | - | + | + | + | | | + | + | | + | + | | + | + |  |
| ***Methylocaldum* sp. SAD2** | + | + | + | + | + | - | + | + | + | | | + | + | | + | + | | + | + |  |

**Supplementary Table S5**. Presence of key pathways/genes involved in methanol, formaldehyde oxidation and assimilation in *Methylocaldum* species with available genomes.


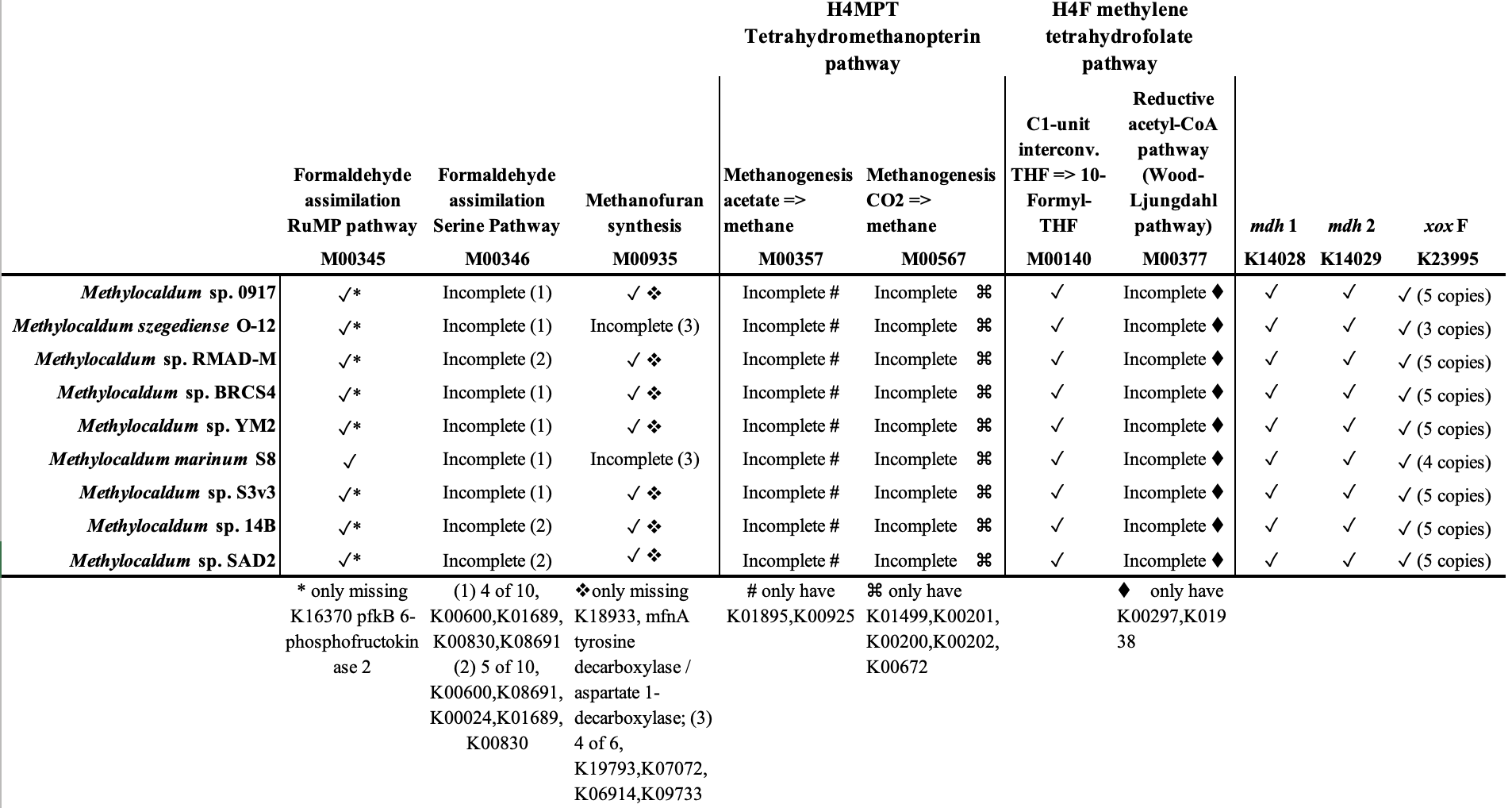


**Supplementary Table S6.** Root-mean-square deviation (RMSD) calculated for *Methylocaldum* PmoC. Measures the RMSD between two sets of atoms in their current positions, without performing any fitting. The RMSD is a commonly used measure of similarity between two protein structures. The smaller the RMSD is between two structures, the more similar are these two structures

| **Strain** | **IMG ID (gene)** | **PmoC Type** | **RMSD (angstroms Å)** | **RMSD (angstroms Å)** |
| --- | --- | --- | --- | --- |
| *Methylocaldum*_S8 | 2832924902 | 1 | 207 pruned atom pairs = 0.749 | All 235 pairs = 1.940 |
| *Methylocaldum*_S8 | 2832927093 | 1 | 207 pruned atom pairs = 0.749 | All 235 pairs = 1.940 |
| *Methylocaldum*_BRCS4 | 2890038637 | 1 | 210 pruned atom pairs = 0.809 | All 235 pairs = 1.795 |
| *Methylocaldum*_14B | 2889962402 | 1 | 213 pruned atom pairs = 0.727 | All 235 pairs = 1.856 |
| *Methylocaldum*_O12 | 2508831462 | 1 | 212 pruned atom pairs = 0.729 | All 235 pairs = 1.877 |
| *Methylocaldum*_O12 | 2508833034 | 1 | 207 pruned atom pairs = 0.735 | All 235 pairs = 1.913 |
| *Methylocaldum*_S8 | 2832925648 | 2 | 211 pruned atom pairs = 0.679 | All 235 pairs = 1.864 |
| *Methylocaldum*_BRCS4 | 2890037627 | 2 | 216 pruned atom pairs = 0.753 | All 235 pairs = 1.472 |
| *Methylocaldum*_RMADM | 2904406329 | 2 | 212 pruned atom pairs = 0.704 | All 235 pairs = 1.822 |
| *Methylocaldum*_14B | 2889959739 | 2 | 212 pruned atom pairs = 0.688 | All 235 pairs = 1.746 |
| *Methylocaldum*_O12 | 2508833640 | 2 | 213 pruned atom pairs = 0.694 | All 235 pairs = 1.859 |
| *Methylocaldum*_O12 | 2508833643 | 2 | 213 pruned atom pairs = 0.732 | All 235 pairs = 1.267 |
| *Methylocaldum*_S3V3 | 2904414129 | 2 | 216 pruned atom pairs = 0.753 | All 235 pairs = 1.472 |
| *Methylocaldum*_BRCS4 | 2890039333 | 3 | 210 pruned atom pairs = 0.685 | All 226 pairs = 1.280 |
| *Methylocaldum*_14B | 2889958190 | 3 | 198 pruned atom pairs = 0.690 | All 231 pairs = 2.715 |
| *Methylocaldum*_O12 | 2508830408 | 3 | 210 pruned atom pairs = 0.716 | All 231 pairs = 1.985 |
| *Methylocaldum*_0917 | 2782372590 | 4 | 219 pruned atom pairs = 0.823 | All 235 pairs = 1.440 |
| *Methylocaldum*_S8 | 2832927990 | 4 | 218 pruned atom pairs = 0.794 | All 235 pairs = 1.817 |
| *Methylocaldum*_BRCS4 | 2890034185 | 4 | 221 pruned atom pairs = 0.778 | All 235 pairs = 1.311 |
| *Methylocaldum*_RMADM | 2904407932 | 4 | 216 pruned atom pairs = 0.797 | All 235 pairs = 1.396 |
| *Methylocaldum*_14B | 2889958847 | 4 | 217 pruned atom pairs = 0.752 | All 235 pairs = 1.408 |
| *Methylocaldum*_YM2 | 2904416963 | 4 | 219 pruned atom pairs = 0.821 | All 235 pairs = 1.434 |
| *Methylocaldum*_S3V3 | 2904412171 | 4 | 219 pruned atom pairs = 0.821 | All 235 pairs = 1.434 |
| *Methylocaldum*_0917 | 2782370918 | 5 | 205 pruned atom pairs = 0.711 | All 236 pairs = 4.370 |
| *Methylocaldum*_S8 | 2832926543 | 5 | 206 pruned atom pairs = 0.731 | All 227 pairs = 1.263 |
| *Methylocaldum*_BRCS4 | 2890037436 | 5 | 209 pruned atom pairs = 0.733 | All 236 pairs = 4.630 |
| *Methylocaldum*_RMADM | 2904406134 | 5 | 209 pruned atom pairs = 0.733 | All 236 pairs = 4.630 |
| *Methylocaldum*_14B | 2889960376 | 5 | 209 pruned atom pairs = 0.733 | All 236 pairs = 4.630 |
| *Methylocaldum*_YM2 | 2904415561 | 5 | 204 pruned atom pairs = 0.692 | All 236 pairs = 4.356 |
| *Methylocaldum*_O12 | 2508833441 | 5 | 211 pruned atom pairs = 0.710 | All 236 pairs = 6.750 |
| *Methylocaldum*_S3V3 | 2904410529 | 5 | 204 pruned atom pairs = 0.692 | All 236 pairs = 4.356 |
| *Methylocaldum*_BRCS4 | 2890034396 |  | 138 pruned atom pairs = 0.834 | All 142 pairs = 0.948 |


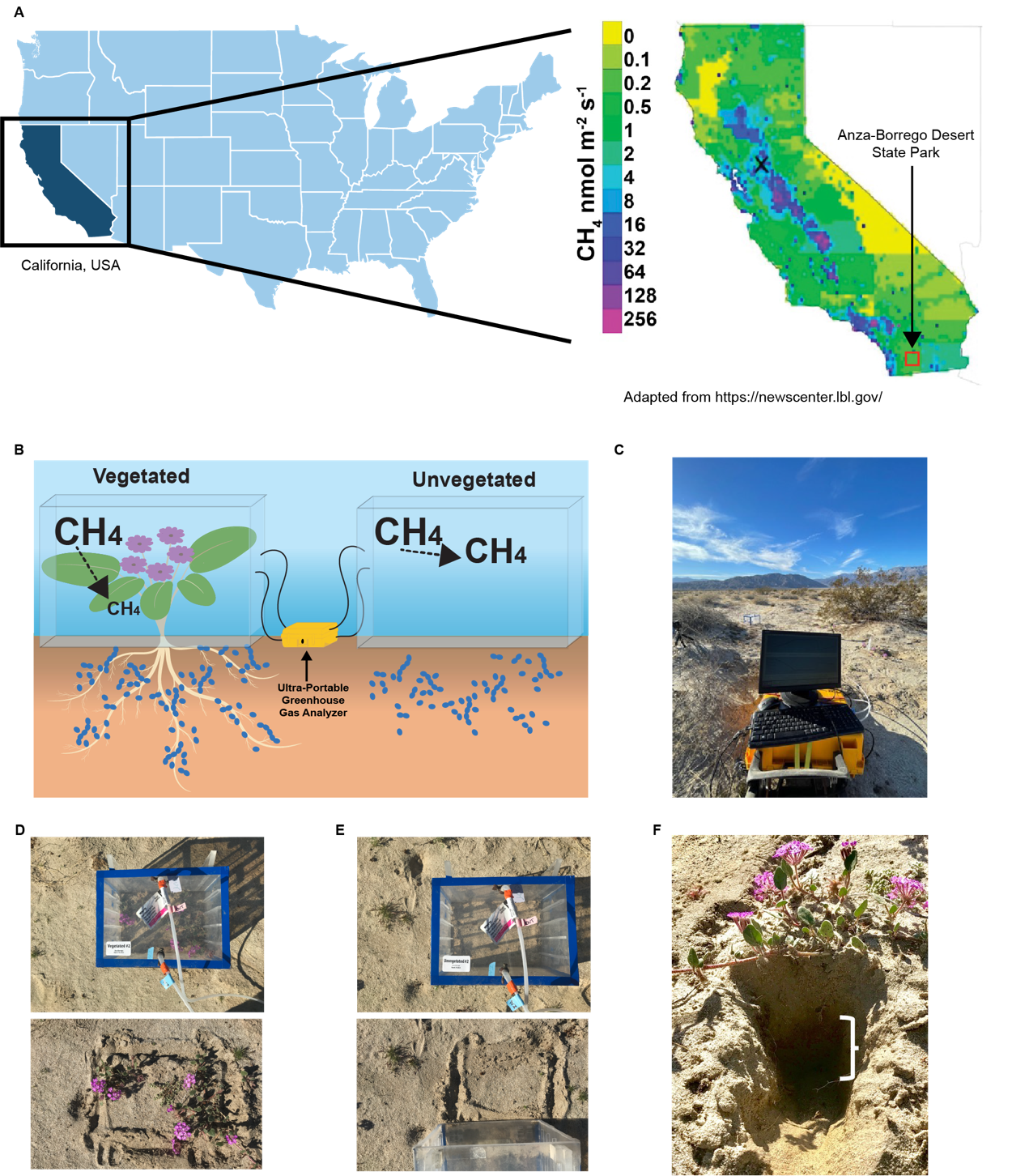


**Supplementary Figure S1.** (A) Methane emissions in California. Location of the Anza Borrego State Park in California, USA. Image adapted from https://newscenter.lbl.gov/. (B) Draw of the experimental setup for in situ measurement of methane fluxes in Anza-Borrego. (C) Photographs of the sampling site and equipment (ultra-portable greenhouse gas analyzer). (D) Photograph of vegetated patch enclosing the endemic plan *Abronia villosa* (desert verbena) with and without acrylic chamber (E) Photographs of unvegetated patch with and without acrylic chamber. (F) The white bracket depicts the depth (10 cm) at which samples were taken under plants. Soil samples for metagenomes, from unvegetated patches were collected at the same depth.

**
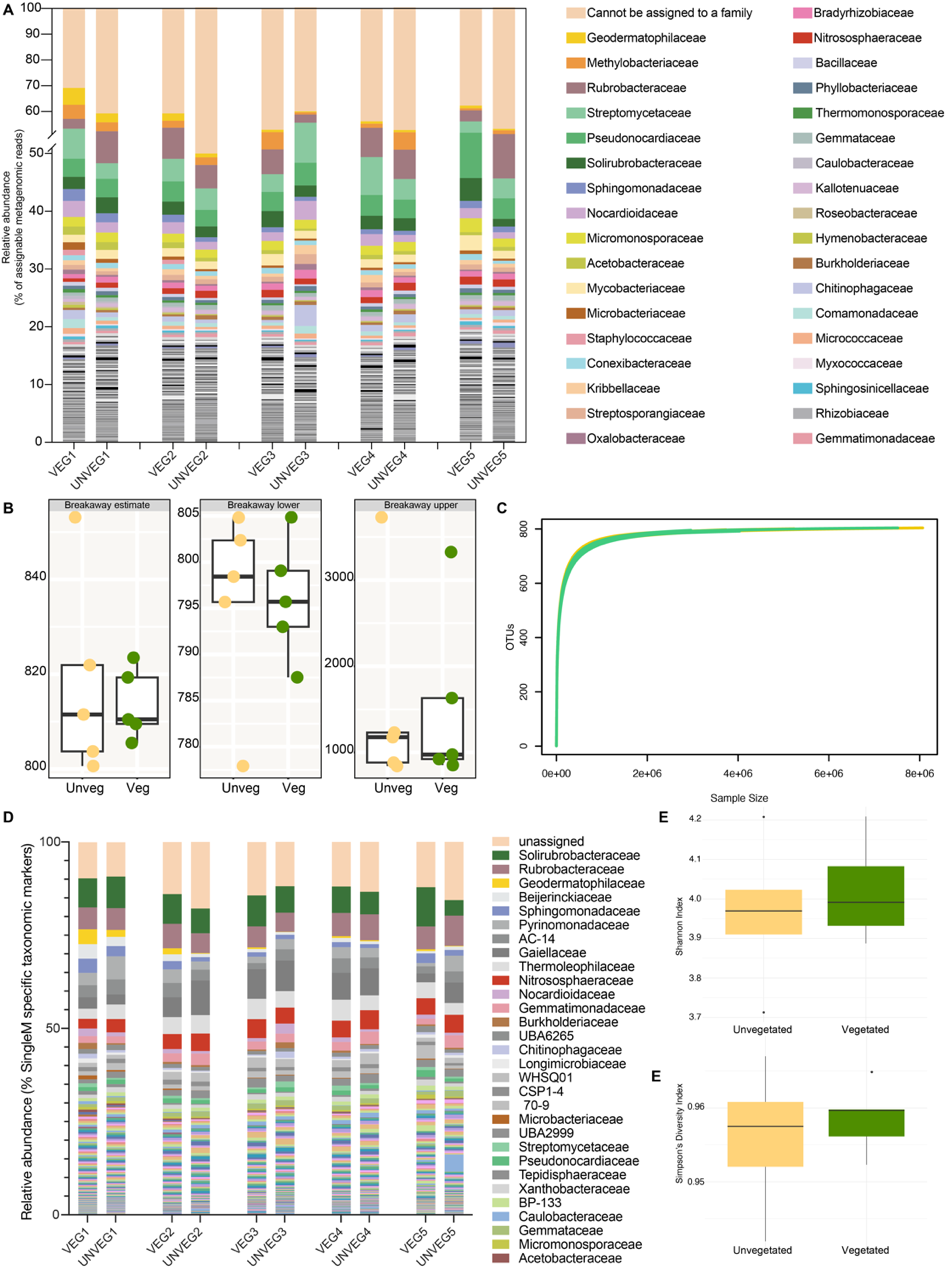
**

**Supplementary Figure S2.** (A) Relative abundance of taxonomically assigned groups at family level obtained with Kaiju. Only abundant families are listed on the legend. (B) Alpha-diversity of raw taxonomically assignable metagenomic reads (Kaiju) of vegetated and unvegetated samples (B) Rarefaction curve. Normalized data was randomly rarefied to 1,721,047 reads. (C) Taxonomic profiling at the family level, based on single-copy marker genes, including uncharacterized lineages (SingleM analysis). (D-E) Alpha diversity was inferred through Shannon and Simpson’s diversity index, based on SingleM taxonomic profiling at the family level.


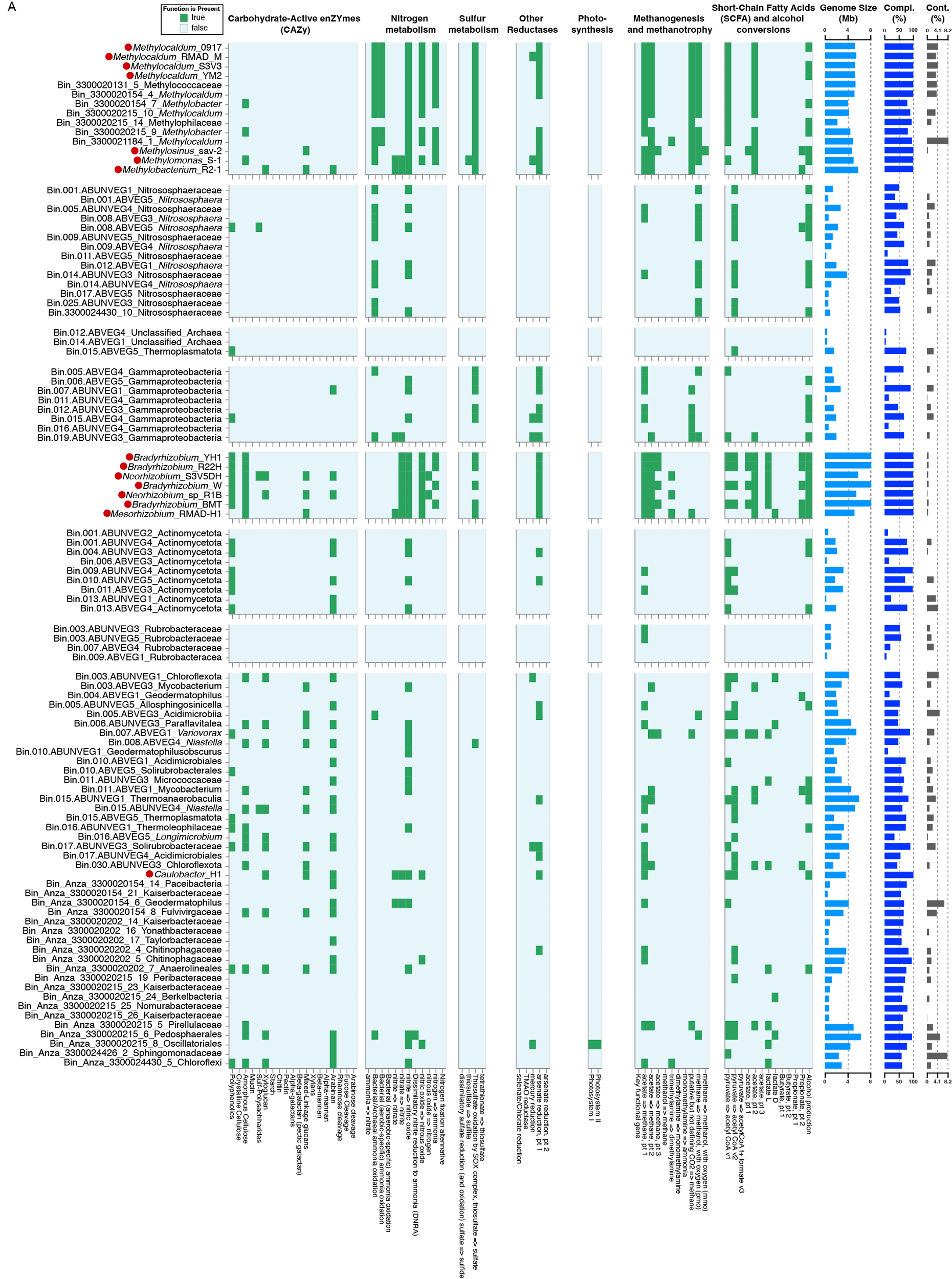


**
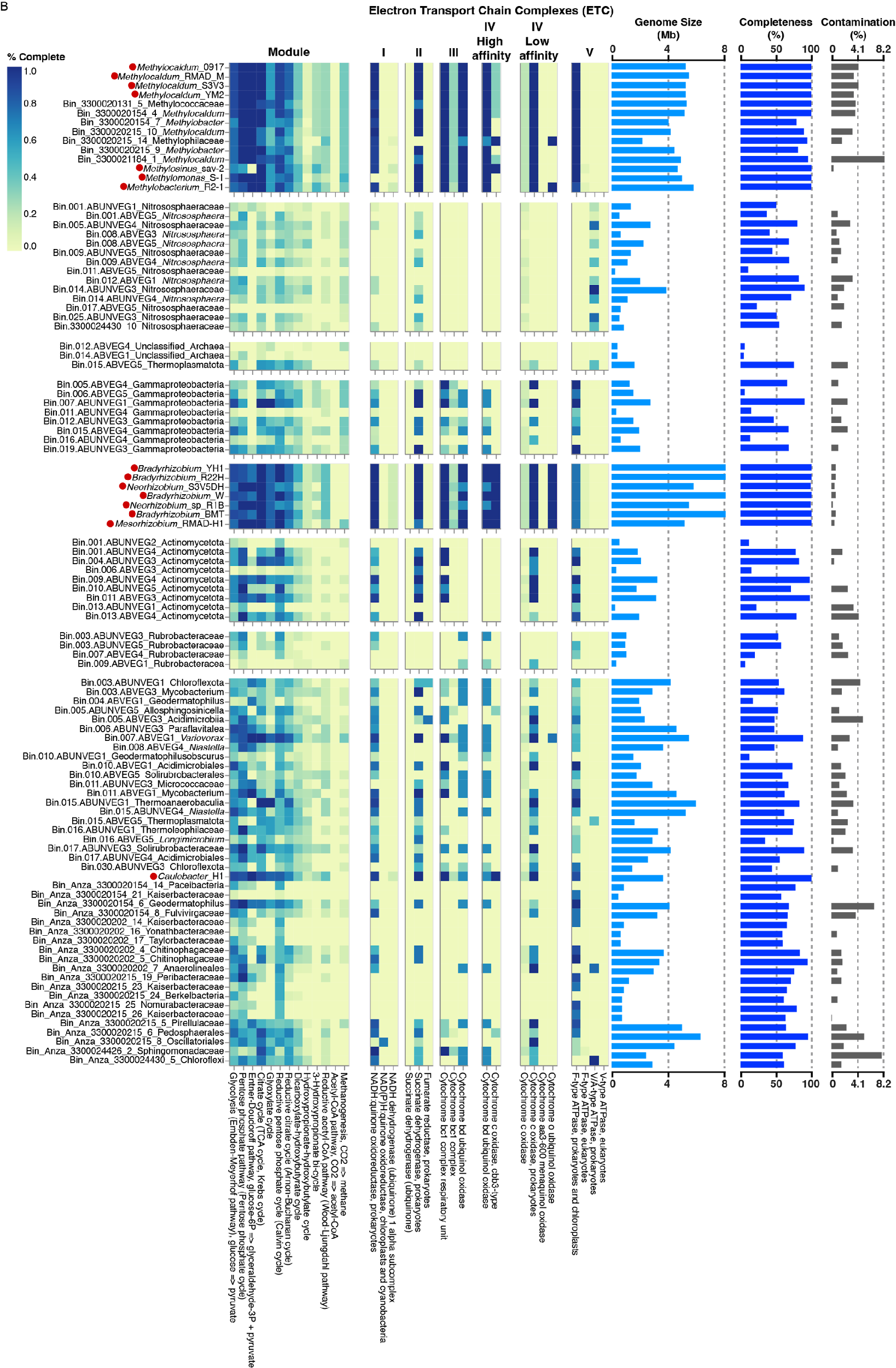
**

**
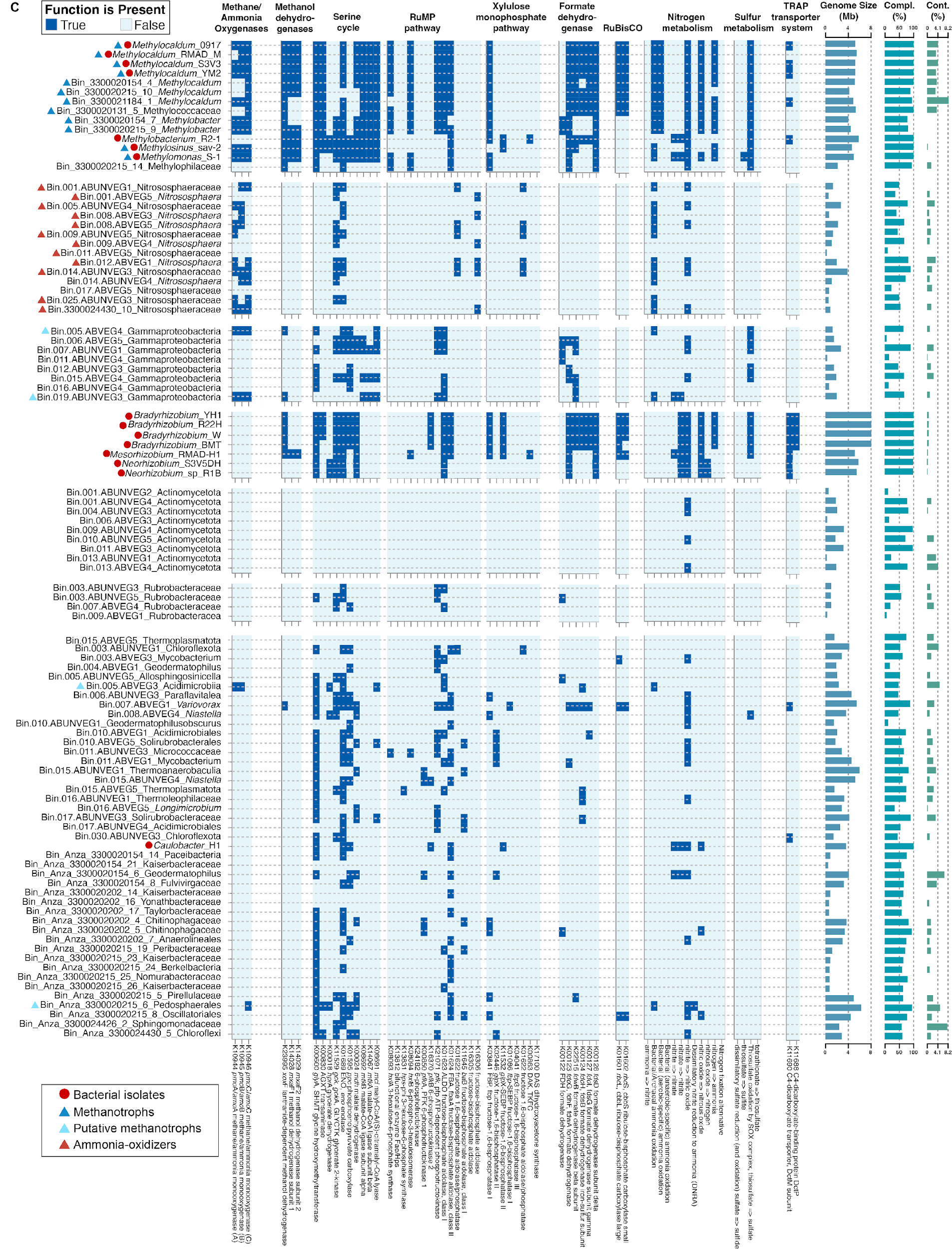
**

**Supplementary Figure S3.** Comparison of the metabolic potential of MAGs generated from Anza-Borrego 2016 and 2023 metagenomes. The analysis indicates the presence of key genes involved in electron transport chain complexes and the (A) Carbohydrate-Active enZYmes (CAZymes). (B) Nitrogen, sulfur, and methane cycling, in addition to short-chain fatty acids (SCFA) metabolism. (C) Comparison of the metabolic potential of isolates and MAGs obtained from Anza-Borrego (2016 and 2023). The analysis indicates the presence of key genes involved in relevant pathways for this study.

**
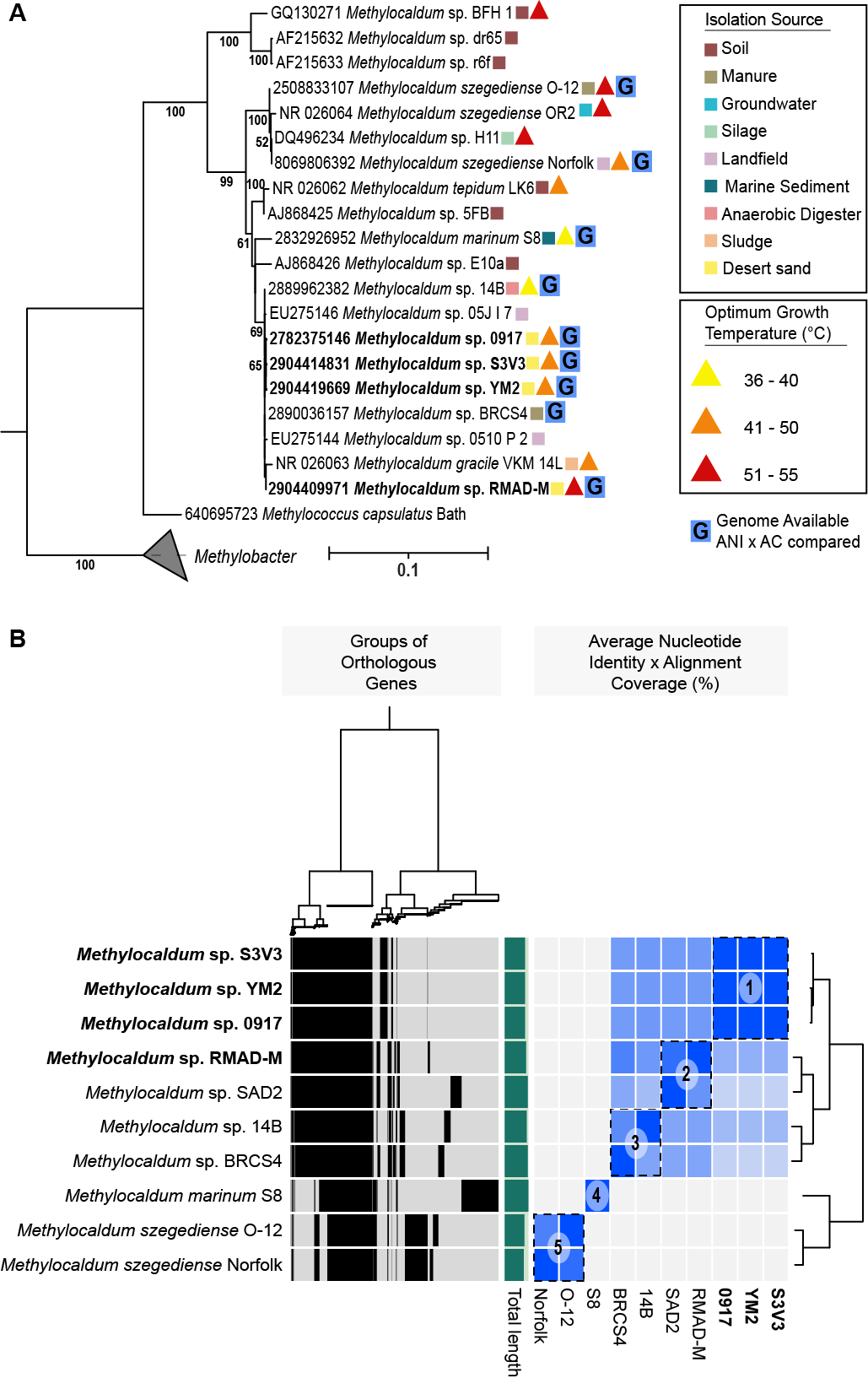
**

**Supplementary Figure S4.** (A) Maximum likelihood tree representing the phylogenetic relationship of *Methylocaldum* based on 16S rRNA gene sequences. The topology of the phylogeny, guided by the presence of type strains, indicates that at least 6 clades are formed within the analyzed strains. Iconography at the end of each leaf indicates the environment from which the strain was isolated, when available, the optimum temperature growth range in laboratory conditions is indicated. (B) Pangenome analysis of the ten available *Methylocaldum* genomes at the time was performed. The examination was based on the distribution of 8,668 groups of orthologous genes (black bars) obtained with Anvi’o. The horizontal bars under General Genome Parameters, represent the values for each of the different parameters measured for each genome. A whole-genome comparison to determine the similitude degree among the strains. For this, a heatmap for Average Nucleotide Identity times the Alignment Coverage (ANI x AC) was generated, on which four major clades can be distinguished.


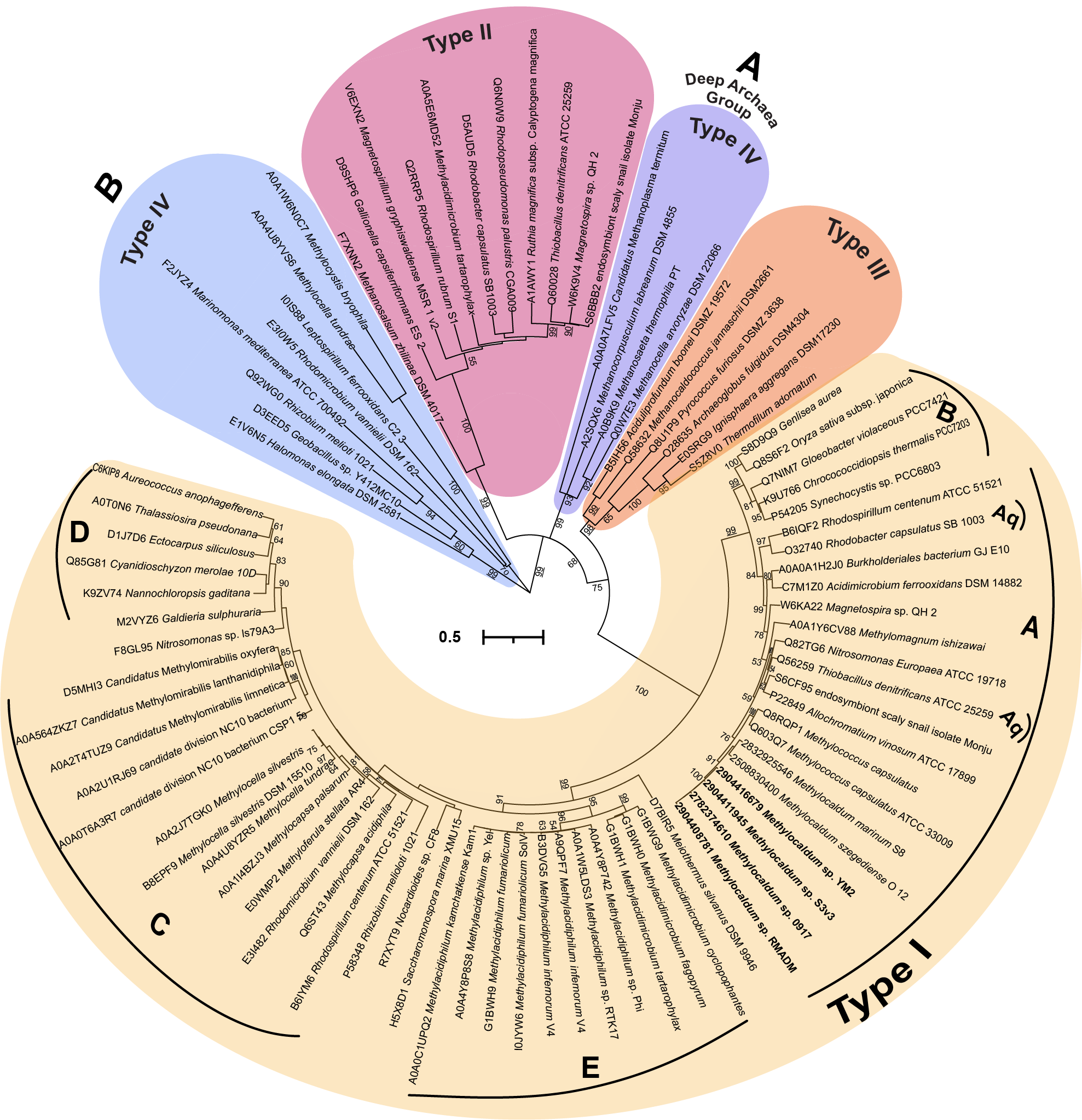


**Supplementary Figure S5**. RuBisCO Tree: Maximum likelihood tree representing the phylogenetic relationship of RuBisCO based on the amino acid sequences of the large subunit gene (*rbcL*) from representative species of the three domains of life.


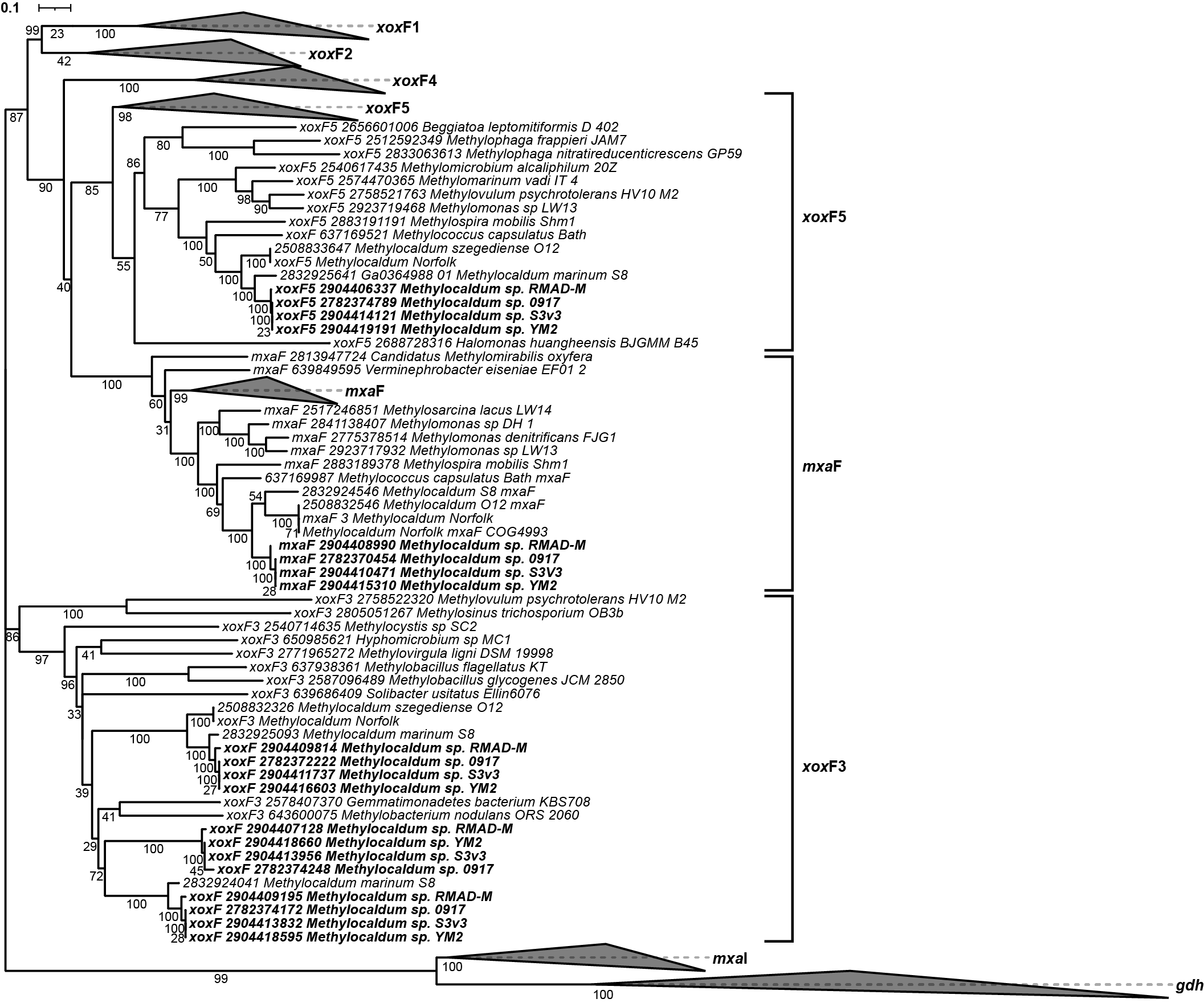


**Supplementary Figure S6**. Phylogenetic reconstruction based on *xox*F and *mxa*F genes. *Methylocaldum* genomes analyzed in this study are highlighted in bold. The corresponding IMG gene IDs are at the tip of each leaf. Collapsed clades did not contain any genes from the three analyzed genomes.


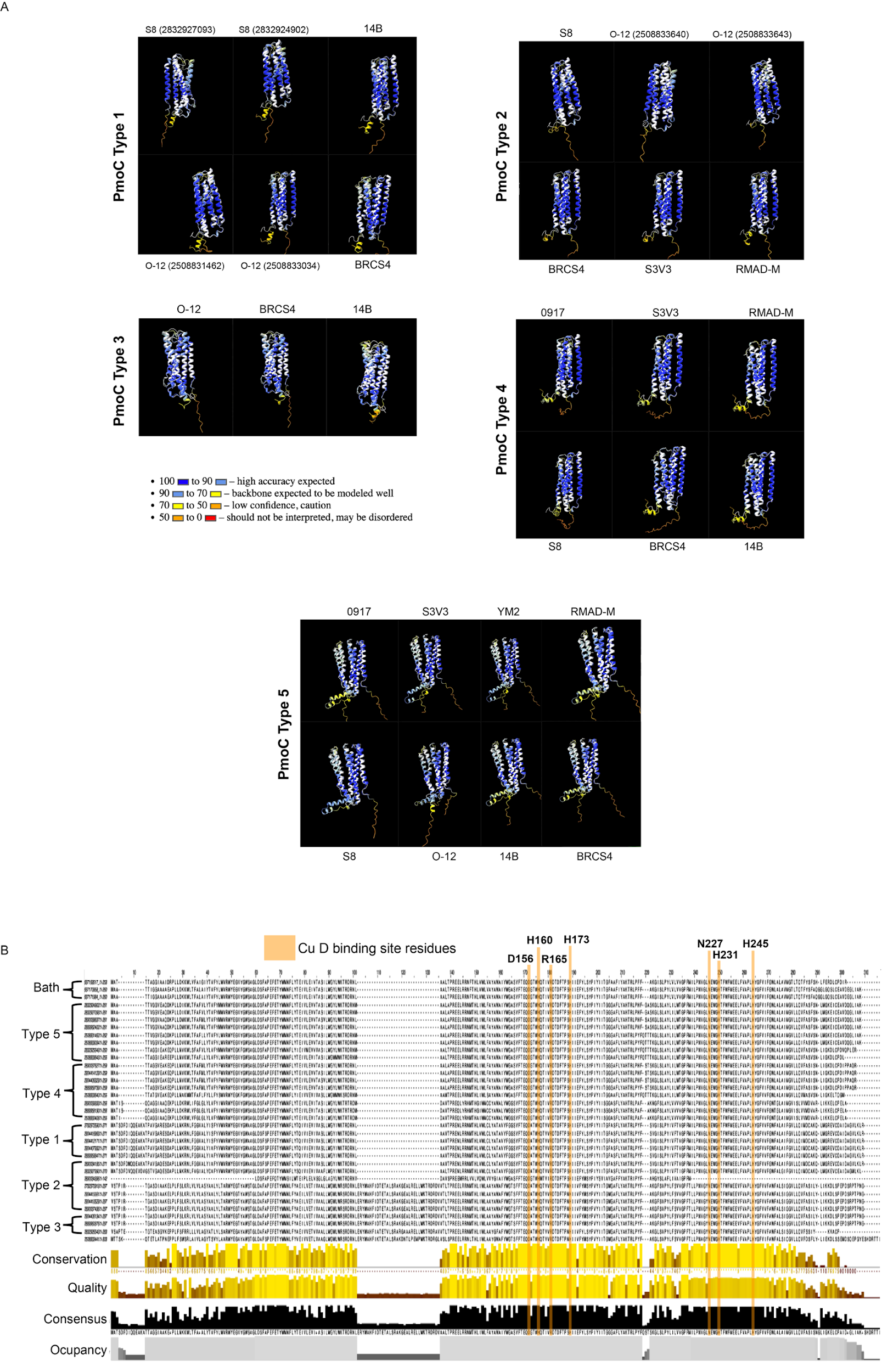


**Supplementary Figure S7.** AlphaFold-predicted tertiary structure of *Methylocaldum*’s PmoC types based on *Methylococcus capsulatus* Bath PmoC crystallography.


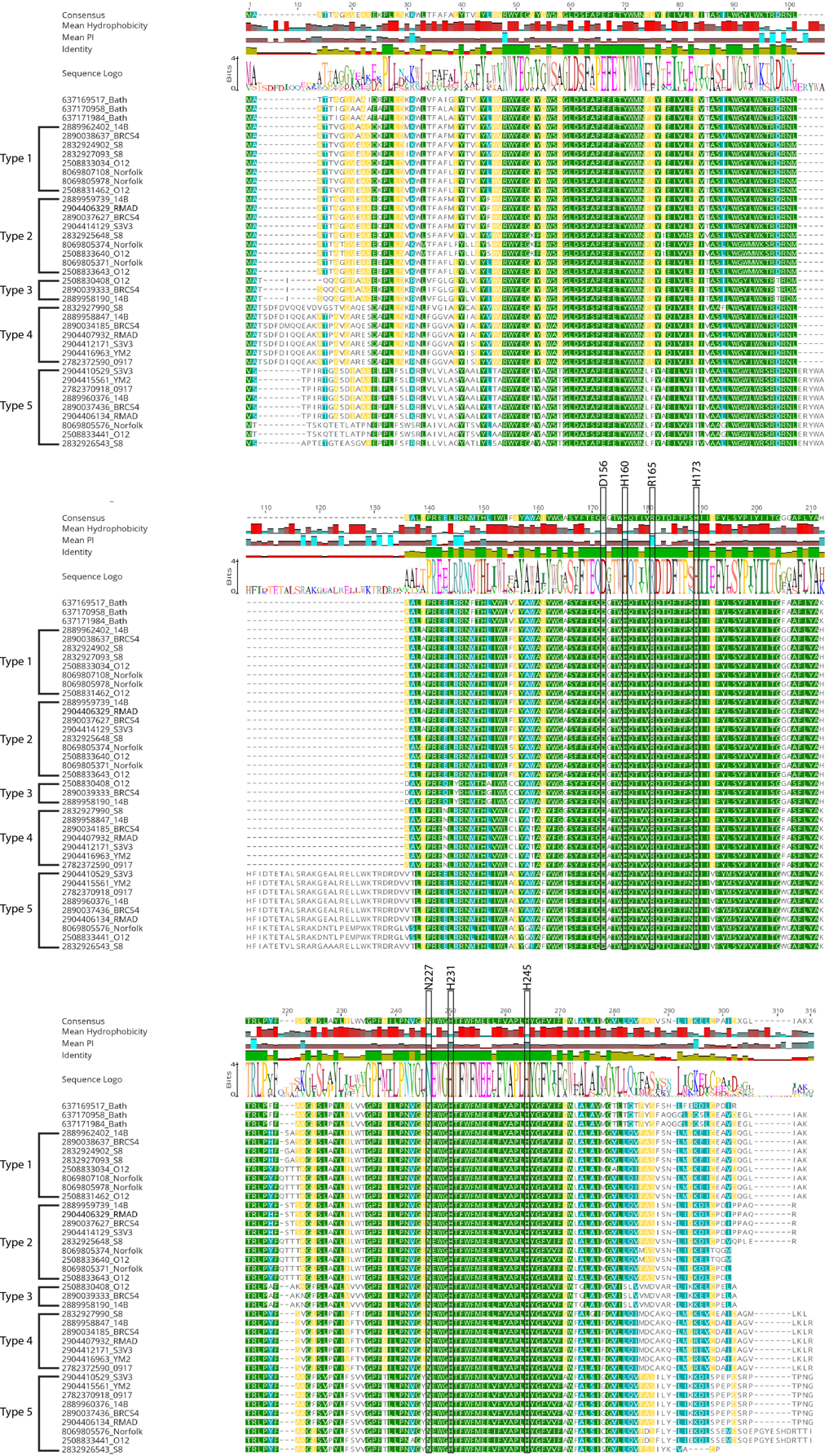


**Supplementary Figure S8.** MAFFT-L-INS-i Alignment for PmoC *Methylocaldum* using MAFFT (v7.511). The amino acid residues forming the Cu D binding site described on Tucci et al., 2023 are highlighted and labeled.


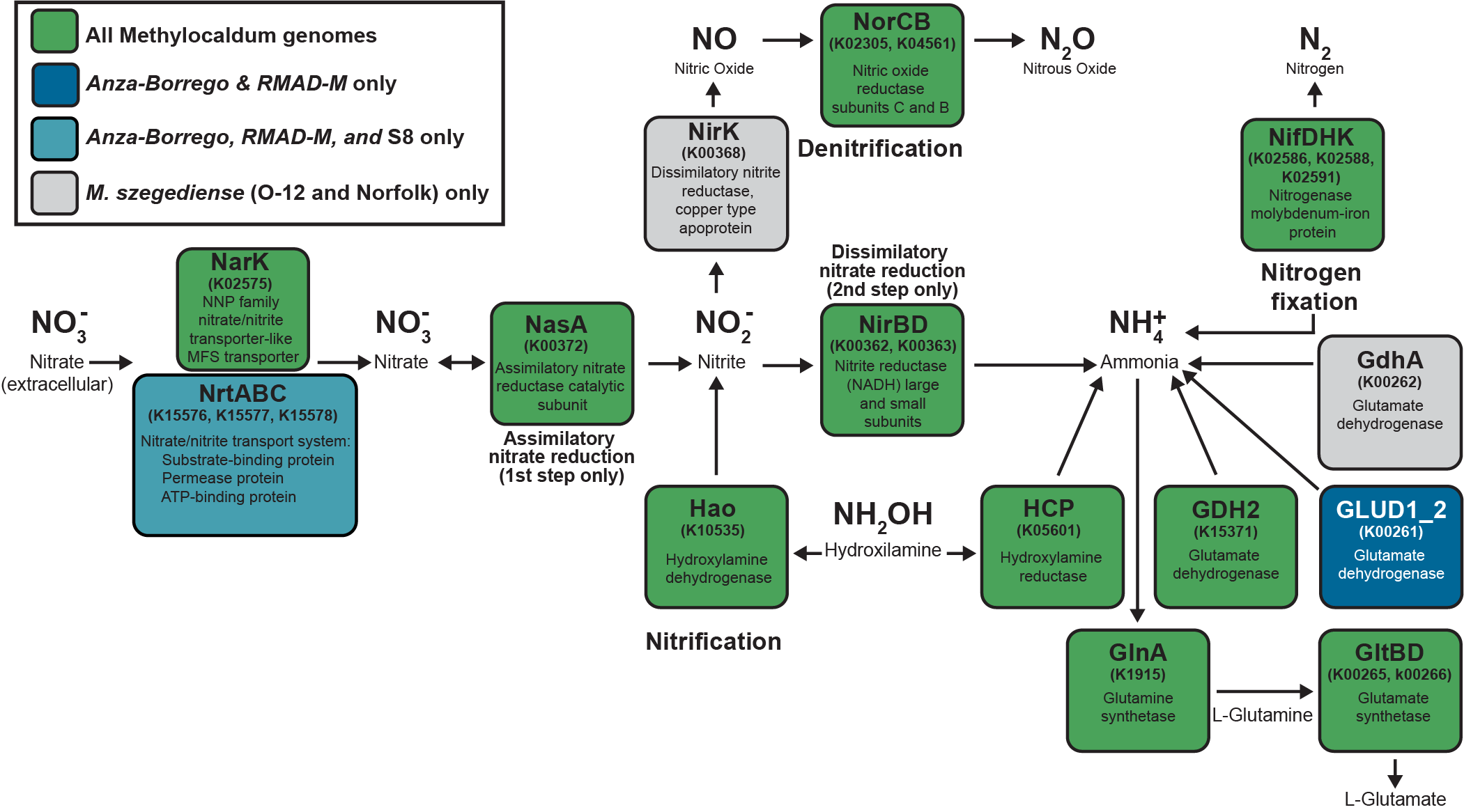


**Supplementary Figure S9.** Nitrogen metabolism genes in *Methylocaldum* strains. Diagram representing the presence and absence of genes involved in nitrogen cycling.

**Supplementary Video S1.** Predicted Alphafold models for PmoC Type 1 (canonical) from *Methylocaldum* strains S8, O-12, 14B, and BRCS4, based on the CryoEM structure of *Methylococcus capsulatus* (Bath) pMMO in a native lipid nanodisc at 2.16 Angstrom resolution (ID 7S4J) [48].

**Supplementary Video S2.** Predicted Alphafold models for PmoC Type 2 from *Methylocaldum* strains S8, O-12, BRCS4, S3V3, and RMAD-M, based on the CryoEM structure of *Methylococcus capsulatus* (Bath) pMMO in a native lipid nanodisc at 2.16 Angstrom resolution (ID 7S4J) [48].

**Supplementary Video S3.** Predicted Alphafold models for PmoC Type 3 from *Methylocaldum* strains O-12, BRCS4, and 14B, based on the CryoEM structure of *Methylococcus capsulatus* (Bath) pMMO in a native lipid nanodisc at 2.16 Angstrom resolution (ID 7S4J) [48].

**Supplementary Video S4.** Predicted Alphafold models for PmoC Type 4 from *Methylocaldum* strains 0917, S3V3, RMAD-M, S8, BRCS4, and 14B, based on the CryoEM structure of *Methylococcus capsulatus* (Bath) pMMO in a native lipid nanodisc at 2.16 Angstrom resolution (ID 7S4J) [48].

**Supplementary Video S5.** Predicted Alphafold models for PmoC Type 5 from *Methylocaldum* strains 0917, S3V3, YM2, RMAD-M, S8, O-12, 14B, and BRCS4, based on the CryoEM structure of *Methylococcus capsulatus* (Bath) pMMO in a native lipid nanodisc at 2.16 Angstrom resolution (ID 7S4J) [48].

**References**

1. Kalyuzhnaya M Methane biocatalysis: Selecting the right microbe. *Biotechnology for biofuel production and optimization*, Elsevier. 353-83

2. Collins DA, Kalyuzhnaya MG. Navigating methane metabolism: Enzymes, compartments, and networks. *Methods Enzymol*. 2018;**613**:349-83 https://doi.org/10.1016/bs.mie.2018.10.010

3. Povedano-Priego C, Jroundi F, Lopez-Fernandez M *et al.* Deciphering indigenous bacteria in compacted bentonite through a novel and efficient DNA extraction method: Insights into biogeochemical processes within the deep geological disposal of nuclear waste concept. *Journal of Hazardous Materials*. 2021;**408**:124600

4. Zeugin JA, Hartley JL. Ethanol precipitation of DNA. *Focus*. 1985;**7**:1-2

5. Naviaux RK, Good B, McPherson JD *et al.* Sand DNA—a genetic library of life at the water’s edge. *Marine Ecology Progress Series*. 2005;**301**:9-22

6. Delherbe N, Pearce D, But SY *et al.* Genomic insights into moderately thermophilic methanotrophs of the genus *Methylocaldum*. 2024

7. He H, Edlich-Muth C, Lindner SN *et al.* Ribulose monophosphate shunt provides nearly all biomass and energy required for growth of *E. coli*. *ACS synthetic biology*. 2018;**7**:1601-11

8. Pham DN, Nguyen AD, Lee EY. Outlook on engineering methylotrophs for one-carbon-based industrial biotechnology. *Chemical Engineering Journal*. 2022:137769

9. Henard CA, Wu C, Xiong W *et al.* Ribulose-1, 5-bisphosphate carboxylase/oxygenase (RuBisCo) is essential for growth of the methanotroph *Methylococcus capsulatus* strain bath. *Applied and environmental microbiology*. 2021;**87**:e00881-21

10. Poudel S, Pike DH, Raanan H *et al.* Biophysical analysis of the structural evolution of substrate specificity in rubisco. *Proceedings of the National Academy of Sciences*. 2020;**117**:30451-57

11. Heureux AM, Young JN, Whitney SM *et al.* The role of RuBisCo kinetics and pyrenoid morphology in shaping the ccm of haptophyte microalgae. *Journal of Experimental Botany*. 2017;**68**:3959-69

12. Semrau JD, DiSpirito AA, Yoon S. Methanotrophs and copper. *FEMS microbiology reviews*. 2010;**34**:496-531

13. Ghosh S, Dhanasingh I, Ryu J *et al.* Crystal structure of cytochrome c_L_ from the aquatic methylotrophic bacterium *Methylophaga aminisulfidivorans* MP^T^. 2020

14. Huang J, Yu Z, Chistoserdova L. Lanthanide-dependent methanol dehydrogenases of *xox*f4 and *xox*f5 clades are differentially distributed among methylotrophic bacteria and they reveal different biochemical properties. *Frontiers in microbiology*. 2018;**9**:1366

15. Sofia HJ, Chen G, Hetzler BG *et al.* Radical sam, a novel protein superfamily linking unresolved steps in familiar biosynthetic pathways with radical mechanisms: Functional characterization using new analysis and information visualization methods. *Nucleic acids research*. 2001;**29**:1097-106

16. Gagsteiger J, Jahn S, Heidinger L *et al.* A cobalamin‐dependent radical SAM enzyme catalyzes the unique C_α_‐methylation of glutamine in methyl‐coenzyme M reductase. *Angewandte Chemie*. 2022;**134**:e202204198

17. Yokogawa T, Nomura Y, Yasuda A *et al.* Identification of a radical SAM enzyme involved in the synthesis of archaeosine. *Nature Chemical Biology*. 2019;**15**:1148-55

18. Hillmeier M, Wagner M, Ensfelder T *et al.* Synthesis and structure elucidation of the human tRNA nucleoside mannosyl-queuosine. *Nature Communications*. 2021;**12**:7123

19. Hori H. Regulatory factors for tRNA modifications in extreme-thermophilic bacterium *Thermus thermophilus*. *Frontiers in genetics*. 2019;**10**:204

20. Holliday GL, Akiva E, Meng EC *et al.* Atlas of the radical SAM superfamily: Divergent evolution of function using a “plug and play” domain. *Methods in enzymology*, Elsevier. 1-71

21. Boswinkle K, McKinney J, Allen KD. Highlighting the unique roles of radical S-adenosylmethionine enzymes in methanogenic archaea. *Journal of Bacteriology*. 2022;**204**:e00197-22

22. Roohi, Zaheer MR, Kuddus M. Phb (poly‐β‐hydroxybutyrate) and its enzymatic degradation. *Polymers for Advanced Technologies*. 2018;**29**:30-40

23. Dibrov P, Dibrov E, Pierce GN. Na+-nqr (na+-translocating NADH: Ubiquinone oxidoreductase) as a novel target for antibiotics. *FEMS microbiology reviews*. 2017;**41**:653-71

24. Xing J, Gumerov VM, Zhulin IB. Origin and functional diversification of PAS domain, a ubiquitous intracellular sensor. *Science Advances*. 2023;**9**:eadi4517

25. Delumeau O, Dutta S, Brigulla M *et al.* Functional and structural characterization of RsbU, a stress signaling protein phosphatase 2C. *Journal of Biological Chemistry*. 2004;**279**:40927-37

26. Makino M, Kondo S, Kaneko T *et al.* Expression, crystallization and preliminary crystallographic analysis of the PAS domain of RsbP, a stress-response phosphatase from *Bacillus subtilis*. *Acta Crystallographica Section F: Structural Biology and Crystallization Communications*. 2009;**65**:559-61

27. Jaiswal RK, Manjeera G, Gopal B. Role of a PAS sensor domain in the *Mycobacterium tuberculosis* transcription regulator Rv1364c. *Biochemical and biophysical research communications*. 2010;**398**:342-49

28. Aravind L, Anantharaman V, Balaji S *et al.* The many faces of the helix-turn-helix domain: Transcription regulation and beyond. *FEMS microbiology reviews*. 2005;**29**:231-62

29. Unden G, Schirawski J. The oxygen‐responsive transcriptional regulator fnr of *Escherichia coli*: The search for signals and reactions. *Molecular microbiology*. 1997;**25**:205-10

30. Mielecki D, Grzesiuk E. Ada response–a strategy for repair of alkylated DNA in bacteria. *FEMS microbiology letters*. 2014;**355**:1-11

31. Karmanova AN, Nikulin NA, Zimin AA. Structural organization, evolution, and distribution of viral pyrimidine dimer-DNA glycosylases. *Biophysical Reviews*. 2022;**14**:923-32

32. Bodrossy L, Holmes EM, Holmes AJ *et al.* Analysis of 16s rRNA and methane monooxygenase gene sequences reveals a novel group of thermotolerant and thermophilic methanotrophs, *Methylocaldum* gen. Nov. *Archives of Microbiology*. 1997;**168**:493-503

33. Romanovskaya V, Malashenko YR, Bogachenko V. Corrected diagnoses of genera and species of methane assimilating bacteria. *Микробиология*. 1978;**47**:120-30

34. Takeuchi M. *Methylocaldum*. *Bergey's Manual of Systematics of Archaea and Bacteria*. 2016:1-5 https://doi.org/https://doi.org/10.1002/9781118960608.gbm01180.pub2

35. Takeuchi M, Kamagata Y, Oshima K *et al.* *Methylocaldum marinum* sp. Nov., a thermotolerant, methane-oxidizing bacterium isolated from marine sediments, and emended description of the genus *Methylocaldum*. *International Journal of systematic and evolutionary microbiology*. 2014;**64**:3240-46

36. Bodrossy L, Murrell JC, Dalton H *et al.* Heat-tolerant methanotrophic bacteria from the hot water effluent of a natural gas field. *Applied and environmental microbiology*. 1995;**61**:3549-55

37. Eshinimaev BT, Medvedkova K, Khmelenina V *et al.* New thermophilic methanotrophs of the genus *Methylocaldum*. *Microbiology*. 2004;**73**:448-56

38. Reddy KR, Rai RK, Green SJ *et al.* Effect of temperature on methane oxidation and community composition in landfill cover soil. *Journal of Industrial Microbiology and Biotechnology*. 2019;**46**:1283-95

39. Sheets JP, Ge X, Li Y-F *et al.* Biological conversion of biogas to methanol using methanotrophs isolated from solid-state anaerobic digestate. *Bioresource technology*. 2016;**201**:50-57

40. Wei X, Ge X, Li Y *et al.* Draft genome sequence of *Methylocaldum* sp. Strain 14b, an obligate hydrogen sulfide-tolerant methanotrophic strain that can convert biogas to methanol. *Genome announcements*. 2017;**5**:e00153-17

41. Su Z, Ge X, Zhang W *et al.* Methanol production from biogas with a thermotolerant methanotrophic consortium isolated from an anaerobic digestion system. *Energy & Fuels*. 2017;**31**:2970-75

42. Wei X, Ge X, Li Y *et al.* Draft genome sequence of *Methylocaldum* sp. SAD2, a methanotrophic strain that can convert raw biogas to methanol in the presence of hydrogen sulfide. *Genome announcements*. 2017;**5**:e00716-17

43. Chang C-Y, Tung H-H, Tseng I-C *et al.* Dynamics of methanotrophic communities in tropical alkaline landfill upland soil. *Applied soil ecology*. 2010;**46**:192-99

44. Deng H, Guo G-X, Zhu Y-G. Pyrene effects on methanotroph community and methane oxidation rate, tested by dose–response experiment and resistance and resilience experiment. *Journal of Soils and Sediments*. 2011;**11**:312-21

45. Islam T, Torsvik V, Larsen Ø *et al.* Acid-tolerant moderately thermophilic methanotrophs of the class gammaproteobacteria isolated from tropical topsoil with methane seeps. *Frontiers in microbiology*. 2016;**7**:851

46. Knief C, Dunfield PF. Response and adaptation of different methanotrophic bacteria to low methane mixing ratios. *Environmental Microbiology*. 2005;**7**:1307-17

47. Tchawa Yimga M, Dunfield PF, Ricke P *et al.* Wide distribution of a novel *pmo*A-like gene copy among type II methanotrophs, and its expression in *Methylocystis* strain SC2. *Applied and environmental microbiology*. 2003;**69**:5593-602

48. Koo CW, Tucci FJ, He Y *et al.* Recovery of particulate methane monooxygenase structure and activity in a lipid bilayer. *Science*. 2022;**375**:1287-9
